# Increased expression of the thyroid hormone nuclear receptor TRα1 characterizes intestinal tumors with high Wnt activity

**DOI:** 10.18632/oncotarget.25741

**Published:** 2018-07-24

**Authors:** Joel Uchuya-Castillo, Nicolas Aznar, Carla Frau, Pierre Martinez, Clementine Le Nevé, Laetitia Marisa, Luiz O.F. Penalva, Pierre Laurent-Puig, Alain Puisieux, Jean-Yves Scoazec, Jacques Samarut, Stephane Ansieau, Michelina Plateroti

**Affiliations:** ^1^ Centre de Recherche en Cancérologie de Lyon, INSERM U1052, CNRS UMR5286, Université de Lyon, Université Lyon 1, Centre Léon Bérard, Département de la recherche, Lyon 69000, France; ^2^ Programme Cartes d’Identité des Tumeurs (CIT), Ligue Nationale Contre le Cancer, Paris 75000, France; ^3^ Children’s Cancer Research Institute, University of Texas Health Science Center at San Antonio, TX 78229, USA; ^4^ UMR-S 1147, Université Paris Descartes, Paris 75006, France; ^5^ Institute Gustave Roussy, Villejuif 94800, France; ^6^ Institute de Génomique Fonctionnelle de Lyon, ENS de Lyon, Lyon 69342, France

**Keywords:** intestinal cancer, thyroid hormone, thyroid hormone nuclear receptor, Wnt antagonist, Wnt pathway

## Abstract

Our previous work demonstrated a key function of the thyroid hormone nuclear receptor TRα1, a T3-modulated transcription factor, in controlling intestinal development and homeostasis *via* the Wnt and Notch pathways. Importantly, increased expression of TRα1 in the intestinal epithelium in a mutated *Apc* genetic background (*vil*-TRα1/Apc^+/1638N^ mice) accelerated tumorigenesis and contributed to a more aggressive tumor phenotype compared to that of the *Apc* mutants alone. Therefore, the aim of this study was to determine the relevance of this synergistic effect in human colorectal cancers and to gain insights into the mechanisms involved. We analyzed cohorts of patients by *in silico* and experimental approaches and observed increased TRα1 expression and a significant correlation between TRα1 levels and Wnt activity. TRα1 loss-of-function and gain-of-function in Caco2 cell lines not only confirmed that TRα1 levels control Wnt activity but also demonstrated the role of TRα1 in regulating cell proliferation and migration. Finally, upon investigation of the molecular mechanisms responsible for the Wnt-TRα1 association, we described the repression by TRα1 of several Wnt inhibitors, including *Frzb*, *Sox17* and *Wif1*. In conclusion, our results underline an important functional interplay between the thyroid hormone nuclear receptor TRα1 and the canonical Wnt pathway in intestinal cancer initiation and progression. More importantly, we show for the first time that the expression of TRα1 is induced in human colorectal cancers.

## INTRODUCTION

The intestinal epithelium is a dynamic tissue that is continuously renewed by stem cells and progenitors located in the crypts of Liberkühn. Its development and homeostasis involve several signaling pathways, including Wnt, Notch and BMP that also exhibit cross-talk with one another [1]; importantly, the dysregulation of these pathways correlates with tumor induction and/or progression [2, 3]. In fact, the ‘adenoma–carcinoma’ sequence occurs through genetic alterations of oncogenes and/or tumor suppressor genes [4] that are also components of the molecular pathways controlling intestinal homeostasis [1]. Thus, symmetry exists between their roles in gut physiology and their roles in tumor initiation and/or progression. This is particularly well characterized in the case of the canonical Wnt pathway, for which the mammalian intestine is one of the most-studied paradigms [5]. Within this context, several investigations demonstrated that thyroid hormone (TH) signaling is also a key regulator of intestinal development and homeostasis in mammals [6]. Strikingly, alteration of TH levels has also been implicated in several cancers, in which hypo- or hyperthyroidism has been linked to a tissue-/organ-specific pro- or anti-tumorigenic function [7]. It is worth noting that, even if a consensus is still lacking, accumulating data underline that TH-signaling plays a tumor-inducing role in the intestine [8].

From a molecular point of view, THs act *via* the thyroid hormone nuclear receptors, the TRs, which are T3-modulated transcription factors [9] belonging to the nuclear hormone receptor protein superfamily [10]. TRs regulate the transcription of target genes, in both a positive and negative manner, by binding to specific DNA sequences named thyroid hormone response elements (TREs) and by recruiting co-factors upon T3 binding [9]. Studies of *Thra* and/or *Thrb* knockout animals showed that the TRα1 nuclear receptor is responsible for TH signaling in the intestinal crypts, where it controls the balance between cell proliferation and cell differentiation through its actions on the Wnt and Notch pathways [6, 11]. In accordance with this important role, the ectopic expression of TRα1 in the intestinal epithelium (*vil*-TRα1 mice) in an *Apc*-mutated background (*vil*-TRα1/Apc^+/1638N^ mice) is responsible for an acceleration of tumor appearance, progression and aggressiveness compared with the *Apc* mutants alone [12]. Hyperactivated Wnt was specifically observed in the double-mutant mice, but the underlying mechanisms involved in the oncogenic synergy remained elusive.

The aim of the current work was to investigate the mechanisms of this synergy and to define the relevance of TRα1 expression levels in human colorectal cancer (CRC) and ultimately the link between TRα1 and the Wnt pathway in this context. By using various cell and molecular approaches, we were able to demonstrate for the first time that the *THRA* gene and the TRα1 nuclear receptor are up-regulated in human CRCs and that one of the mechanisms involved in regulating Wnt activity in these tumors relies on a TRα1-linked strong inhibition of Wnt antagonists.

## RESULTS

### TRα1 expression is up-regulated in human colorectal cancer patients and is correlated with Wnt/β-catenin activity

**Figure 1:**
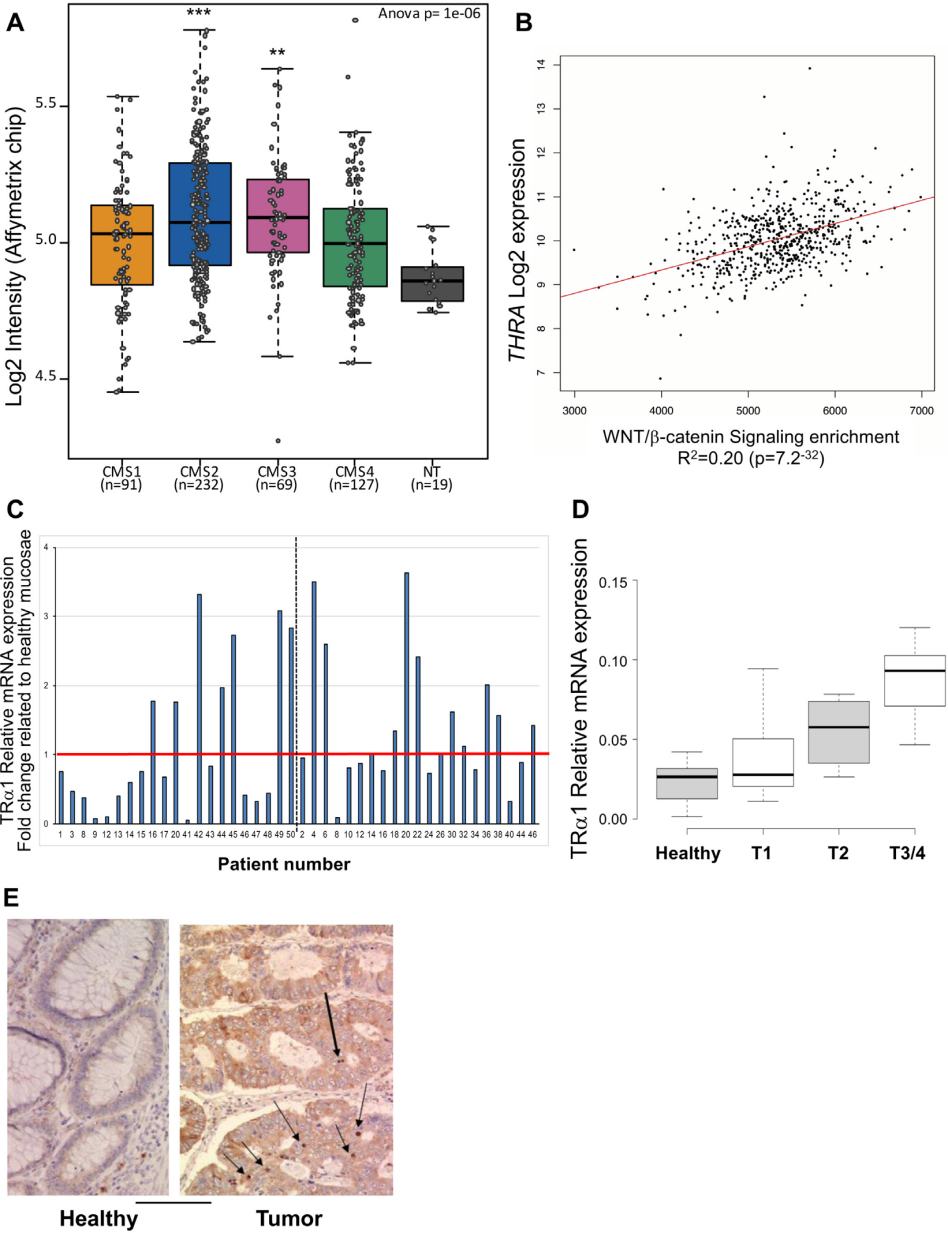
Correlation between TRα1 and Wnt in human colorectal cancer samples (**A**) THRA gene expression levels was evaluated in the GSE39582 dataset [52] and presented as boxplots according to the four-subtypes of the consensus molecular classification of CRC [13]. Note the dispersed expression of *THRA* in the different groups compared with the healthy mucosae (NT). ^**^P = 0.0021 and ^***^P = 0.00028 by ANOVA followed by Tukey’s post hoc test. (**B**) Positive correlation between *THRA* expression and the Wnt signaling pathway in human CRC. *THRA* expression values and Wnt/β-catenin signaling enrichment were analyzed in CRC using the TCGA dataset. The graph shows a highly significant (*P*-value, 7.2 × 10^–32^) direct correlation between *THRA* expression levels and Wnt activity. (**C**) Analysis of TRα1 mRNA expression in cohorts of tumors. Histogram displays TRα1 mRNA expression in each tumor represented as fold change relative to the healthy mucosa of the same patient (expression value in healthy mucosa = 1). The red line delineates over- or unchanged/low-TRα1-expressing tumors; the dotted black line distinguishes the two cohorts analyzed. (**D**) Analysis of TRα1 mRNA expression in healthy mucosae and tumors illustrated in (C) organized according to the increase in tumor stage (from T1 to T4) as indicated. Boxplots show the distribution of data and the mean (black thick line). (**E**) Immunohistochemical analysis of TRα1 expression in healthy mucosa and in cancer. TRα1 appears to be clearly expressed in the nuclei of some epithelial cells in cancer but is not-detectable in normal tissue. Bar, 5 µm.

To investigate the relevance of our mouse *in vivo* data for human CRC patients, we explored the molecular groups defined in Guinney *et al.* [13] and the TCGA database (http://tcga-data.nci.nih.gov/docs/publications/tcga/) to analyze the expression levels of the *THRA* gene *in silico*. In particular, the consensus molecular subtypes (CMSs), representing the most robust classification system currently available for CRC, distinguish four molecular groups: CMS1 (hypermutated, microsatellite unstable and strong immune activation), CMS2 (epithelial, high Wnt and Myc signaling activation), CMS3 (epithelial and metabolic dysregulation) and CMS4 (strong transforming growth factor-β activation, stromal invasion and angiogenesis). Interestingly, *THRA* expression was widely dispersed across the different CMS subtypes (Figure 1A). Nevertheless, all of the groups significantly overexpressed *THRA,* and the most significant overexpression compared with normal tissues was observed in CMS2 and CMS3 (Figure 1A). As mentioned above, these two groups are related to Wnt activity and with metabolic pathways, consistent with our previous results in mice on the association between TRα1 and Wnt [14] and with the well-known action of thyroid hormones/TRs on the metabolism [15]. Interestingly, in TCGA colorectal tumors, the levels of *THRA* expression were significantly and directly correlated with Wnt activity (Figure 1B), once again reinforcing the link between TRα1 and the Wnt pathway. To experimentally validate and specifically study the expression of the TRα1 receptor in human CRCs at the mRNA level, we analyzed two cohorts of patients, including healthy mucosae and cancer samples from each patient (Figure 1C). As expected from the *in silico* studies, we observed some heterogeneity in TRα1 expression levels in tumors compared with their respective healthy mucosae, though approximately 40% of tumors presented increased TRα1 mRNA expression. Moreover, TRα1 up-regulation was correlated with a more advanced tumor stage (Figure 1D). Immunohistochemical analysis revealed a heterogeneous pattern of TRα1 protein expression, but we could clearly visualize TRα1-expressing nuclei, while TRα1 was not detectable in normal counterparts (Figure 1E).

Altogether, these results show that TRα1 is up-regulated in human CRC and is directly correlated with Wnt pathway activation.

### TRα1 expression levels control colon adenocarcinoma cell phenotype *in vitro*

**Figure 2:**
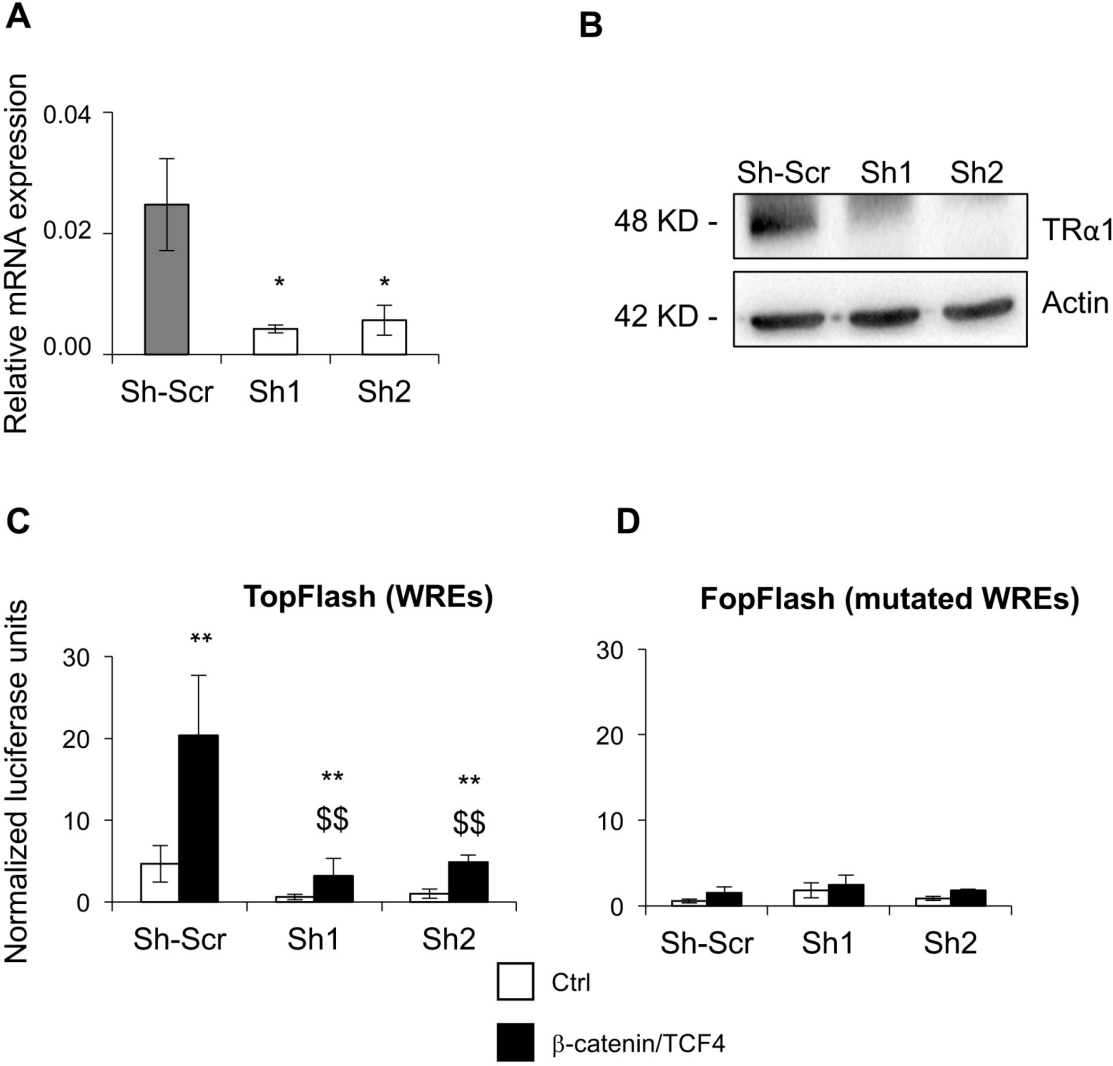
TRα1 enhances b-catenin/TCF4 activity in colorectal cancer cells (**A**, **B**) Generation of TRα1-depleted Caco2 cell lines. Knock-down efficiency was assessed by comparing TRα1 mRNA by RT-qPCR (A) or protein by IB (B). Two independent shRNA sequences targeting TRα1 (efficiency ∼80%) and a scrambled-Sh (Sh-Scr) were used in all of the experiments. Histogram represents the mean ± SD (*N =* 6) from one of three independent experiments. ^*^P < 0.05 compared with Sh-Scr by Student's *T*-test. (**C**, **D**) Depletion of TRα1 decreases canonical Wnt signaling activity. Bar graphs display quantification of luciferase activity in Caco2 cell lines generated in (A) after transfection with the TopFlash vector (C) or the negative control FopFlash vector (D) to monitor Wnt activity by using the Dual-Luciferase Reporter Assay System. Caco2 cells were maintained in culture medium containing physiological concentrations of T3 in the absence (Ctrl) or presence of co-transfected β-catenin/TCF4 complex. Histograms represent the mean ± SD (*N =* 6) from one of three independent experiments. ^**^P < 0.01 compared with the control condition in the same cell line; ^$$^*P <* 0.01 compared with the β-catenin/TCF4 condition in Sh-Scr cells.

To investigate the role of TRα1 in the modulation of key cellular phenotypes regulated by Wnt signaling during tumorigenesis, *i.e.,* cell proliferation/growth and migration, we used Caco2 adenocarcinoma cells. These cells endogenously express TRα1 and have previously been used to study the function of TRα1 in a human colon cancer context [16]. The parental cell line was engineered *via* lentiviral infection of TRα1 expression or silencing (shRNA) vectors in order to develop TRα1 gain-of-function or loss-of-function Caco2 cell lines. We confirmed the efficiency of two shRNAs (named Sh1 and Sh2) against TRα1 and of TRα1 overexpression by RT-qPCR and immunoblot (IB), respectively (Figure 2A and 2B; Supplementary Figure 1A and 1B). Furthermore, the T3/TRα1-dependent responsiveness of the different cell lines was validated using a synthetic TRE-luciferase reporter system (Supplementary Figure 2A and 2B).

First, we investigated whether TRα1 expression levels could modulate Wnt activity in the different Caco2 cell lines using the TopFlash Wnt-reporter assay. As expected, in control cells (Sh-Scr) co-transfection with β-catenin/TCF4 and TopFlash significantly increased the luciferase activity compared with control cells (Ctrl). Both Sh1 and Sh2 showed a strong and significant decrease in TopFlash-dependent Wnt activity under both control and β-catenin/TCF4 conditions (Figure 2C), while TRα1 overexpression, conversely, potentiated Wnt activity (Supplementary Figure 1C). When using the FopFlash vector harboring mutated Wnt response elements, we noted that luciferase activity was not modulated by alterations of TRα1 expression levels, highlighting the specificity of the response (Figure 2D; Supplementary Figure 1D).

**Figure 3:**
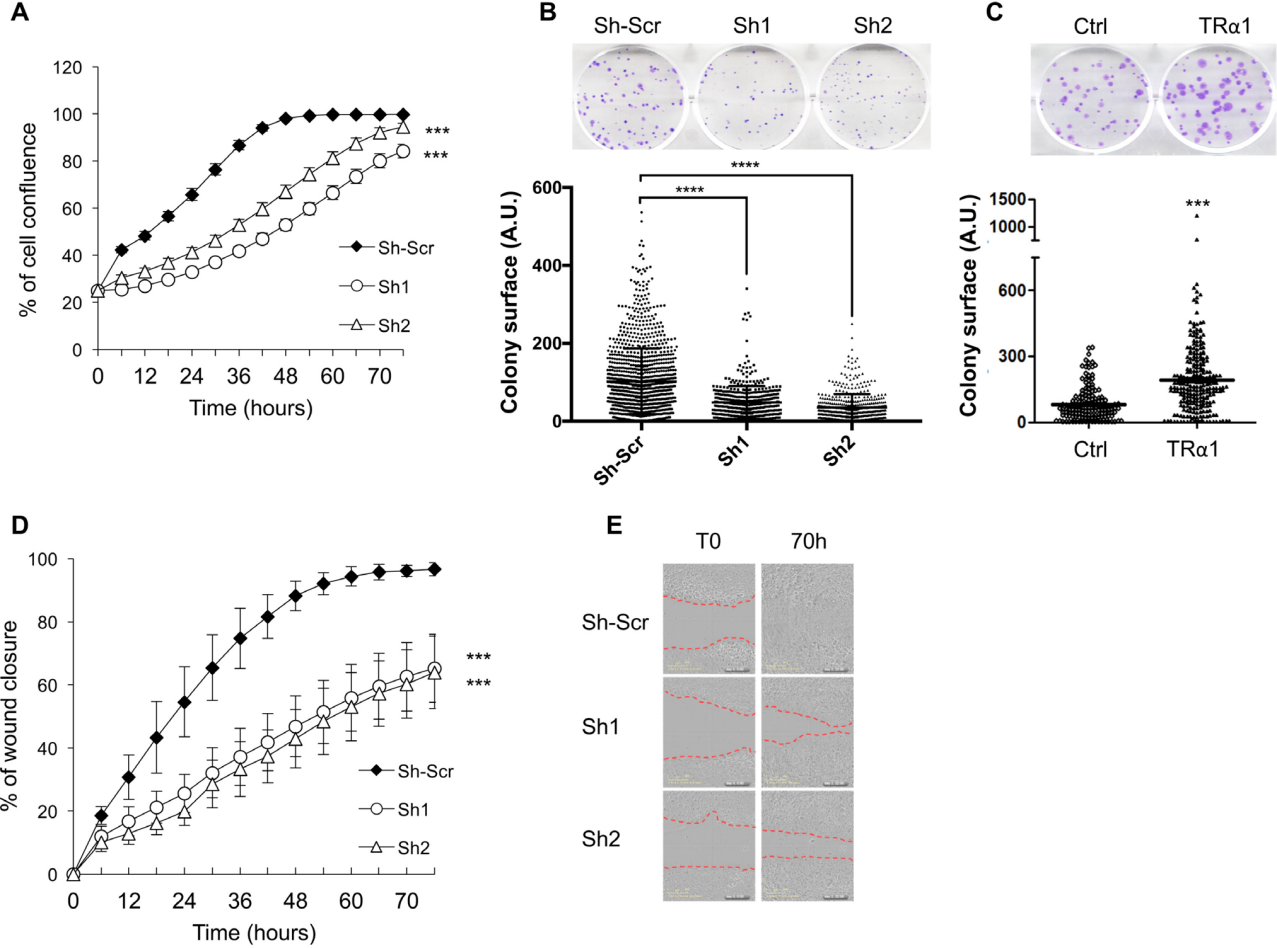
TRα1-dependent cell growth and migration in colorectal cancer cells (**A**) Depletion of TRα1 inhibits cell proliferation. Caco2 cell lines were analyzed for their ability to proliferate during 70 h in culture medium supplemented with 10% FBS. The confluence indexes (y-axis) of the cell lines are displayed as line graphs. Depletion of TRα1 significantly delays cell proliferation. Histogram represents the mean ± SEM (*N =* 10) from one of three independent experiments. (**B**) Depletion of TRα1 inhibits anchorage-dependent cell growth. TRα1-depleted Caco2 cells were analyzed for their ability to form adherent colonies on plastic plates over the course of 2 weeks prior to fixation and staining with crystal violet. The upper panel shows a photograph of the crystal violet-stained 6-well plates. The number of colonies was counted by ImageJ (colony counter), and plot graphs (lower panel) displaying the size (area) of colonies formed (y-axis) for each cell line. (**C**) Increased expression of TRα1 potentiates anchorage-dependent cell growth. TRα1-engineered Caco2 cells were analyzed for their ability to form adherent colonies on plastic plates during 2 weeks prior to fixation and staining with crystal violet. In the upper panel, photograph of the crystal violet-stained 6-well plate is displayed. The number of colonies was counted by ImageJ (colony counter) and the plot shown in the lower panel displays the size (area) of colonies formed (y axis) for each cell line. ^***^P < 0.001 compared with the control cell line by Student’s *T*-test. (**D**, **E**) Depletion of TRα1 inhibits 2-D cell migration. Confluent monolayers of Caco2 cells were scratch-wounded and incubated for 70 h in culture medium supplemented with 10% FBS, and wound closure was monitored and quantified. In panel D, confluence indexes (y-axis) in cell lines are displayed as line graphs. Note that depletion of TRα1 significantly delays wound closure. Histograms represent mean ± SEM (*N =* 10) from one of three independent experiments, each conducted in 10 replicates. Panel E shows representative pictures of different cell lines taken at T0 or T70. Red dashed lines in E delineate the wounded surface (T0) and the surface still depleted of cells (T70). ^***^P < 0.001 and ^****^P < 0.0001 by Student’s *T*-test. ^***^ in A and D applies to each time-point.

Next, we analyzed whether TRα1 alterations could affect key cellular phenotypes regulated by Wnt signaling. Importantly, depletion of TRα1 inhibited, while TRα1-transgenic expression increased, anchorage-dependent cell growth/cell proliferation (Figure 3A–3C). Depletion of TRα1 also inhibited cell migration in *in vitro* wound healing experiments, in both proliferative (FBS-supplementation, Figure 3D and 3E) and non-proliferative (FBS-deprivation, Supplementary Figure 3) conditions. Finally, in the TRα1 transgenic cell line, cell migration was not affected (not shown), probably due to the endogenous TRα1 expression in these cells.

Altogether, these findings demonstrate that TRα1 levels not only positively regulate the canonical Wnt signaling pathway in colon cancer cells but also affect cell proliferation and cell migration.

### TRα1-dependent molecular features of murine intestinal adenocarcinomas

To gain a molecular insight into the mechanisms related to TRα1-dependent tumor phenotype, we took advantage of our murine models. For this aim, the expression profiles of adenocarcinomas from *vil*-TRα1/Apc^+/1638N^ *versus* Apc^+/1638N^ mice (hereafter, *vil*-TRα1/Apc and Apc, respectively) were compared *via* transcriptomic analysis. Of note, the different animals displayed physiological levels of circulating thyroid hormones (Supplementary Table 1).

We used two statistical approaches to identify significantly differentially expressed genes (Supplementary Figure 4A) and retained only the genes pinpointed by both approaches for subsequent analyses (Supplementary Figure 4A; Supplementary Table 2). The hierarchical clustering clearly grouped the genes depending on the tumor genotype (Supplementary Figure 4B), while the Ingenuity Pathway Analysis (IPA) identified biological functions that were significantly associated with the dataset, which included 1) cellular growth and proliferation, 2) cellular development and 3) cancer (Supplementary Figure 5A). IPA also resulted in the identification of the most significant canonical pathways within the dataset (Supplementary Figure 5B). Interestingly, these were the Cdc42, which is related to cell division and cell cycle, and the Wnt/β-catenin pathway, previously correlated with the function of TRα1 [14]. To corroborate the microarray data, we used an RT-qPCR approach and validated most of the differentially expressed genes (DE) that belonged to the Wnt and Notch pathways, to nuclear hormone receptor signaling, or that were strongly regulated (Supplementary Table 3). Interestingly, the top up-regulated genes (Supplementary Figure 6A; Supplementary Table 3) encode proteins involved in tumor progression (Cxcr5 and Aicda), while the top down-regulated genes are strong tumor inhibitors (Anxa10 and Expi) [17–22]. This result is in accordance with more aggressive tumors derived from *vil*-TRα1/Apc mice. Finally, bioinformatics analysis revealed a "Cellular Growth, Cell Proliferation and Cancer" network in which 26 relevant DE genes encoding membrane channels, are linked to cell movement and/or calcium flux and are associated with the Wnt pathway (Supplementary Figure 6B, Supplementary Table 4). In particular, we found that agonists of the Wnt pathway were up-regulated and Wnt antagonists were down-regulated in *vil*-TRα1/Apc tumors (Supplementary Figure 6C). The decrease in Wnt inhibitors in *vil*-TRα1/Apc lesions represented an unexpected novelty that compelled us to preferentially focus on them for further in depth analysis.

### Wnt inhibitors are strongly down-regulated in TRα1-overexpressing tumors

We then decided to analyze *Frzb*, *Sox17* and the Wnt-inhibitory factor 1 (*Wif1*) and examine the relevance of their down-regulation in TRα1-overexpressing tumors. All of them have previously been described as negative regulators of Wnt activity [23–25]. Therefore, their strong repression in TRα1-overexpressing tumors could provide a rationale for the increase in Wnt activity observed in these tumors [12].

**Figure 4:**
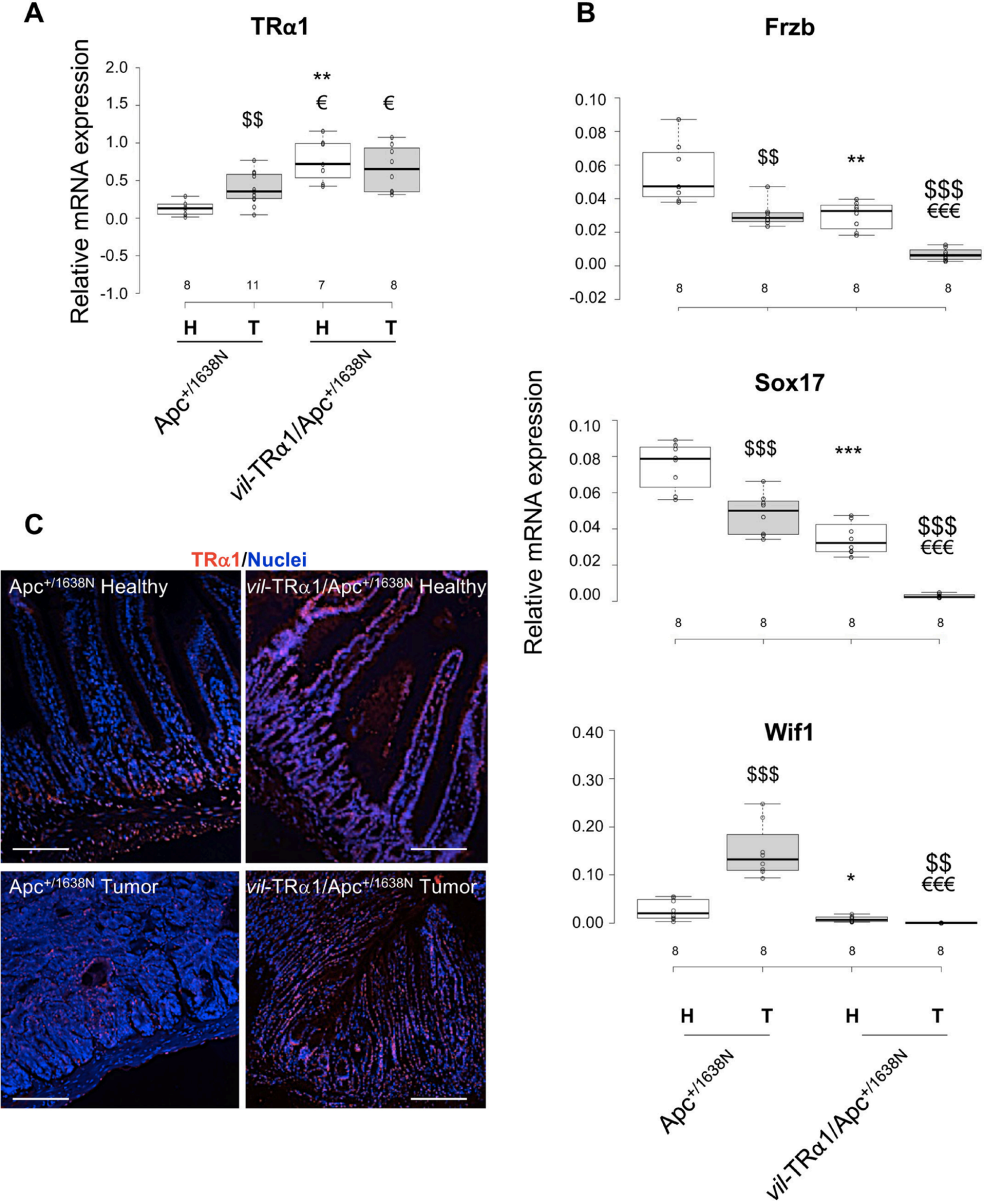
Inverse correlation between TRα1 and Wnt antagonists (**A**, **B**) Analysis of TRα1 mRNA expression (A) and *Frzb*, *Sox17* and *Wif1* mRNA expression (B) in healthy mucosae (H) and tumors (T) from animals of the indicated genotypes. The number of samples analyzed is indicated on the X axis. Boxplots illustrate the distribution of data and the mean (black thick line). Note that *Frzb*, *Sox17* and *Wif1* mRNA expression are down-regulated in *vil*-TRα1/Apc compared to Apc tumors, thus validating the microarray data. ^*^P < 0.05 and ^**^0.01, compared with healthy Apc mucosae; ^$^*P <* 0.05 and ^$$^*P <* 0.01, compared with tumor of the same genotype; ^€^*P <* 0.05 and ^€€^*P <* 0.01, compared with Apc tumors by Student’s *T*-test. **(C)** TRα1 immunolabeling of intestinal sections (healthy mucosa and tumor) from Apc and *vil*-TRα1/Apc mice as indicated. The images, which are representative of at least three tumors per genotype, show merged nuclear staining (blue) and TRα1 staining (red). Bars, 10 µm.

We measured the mRNA expression of TRα1 and each of the Wnt antagonists in normal mucosae and tumors from Apc and *vil*-TRα1/Apc mice (Figure 4A and 4B). As expected, *vil*-TRα1/Apc mice displayed an increase in TRα1 mRNA expression in both healthy mucosae and tumors, significantly higher than in Apc mice (normal tissue or tumors) (Figure 4A). Interestingly, and in line with the results in human CRCs, Apc tumors showed a significant increase in TRα1 expression compared with normal Apc mucosae (Figure 4A). The analysis of TRα1 protein levels by IF confirmed that in healthy Apc mucosae, TRα1 is expressed mainly in crypt cells and in the external muscle layers, as already described [12] (Figure 4C). In addition, we observed a higher percentage of TRα1-expressing nuclei within Apc tumors compared to their normal counterparts. Finally, we also revealed the presence of TRα1 in all of the epithelial cells of *vil*-TRα1/Apc mice, consistent with the TRα1-transgenic expression in these cells (Figure 4C). Regarding the expression of the Wnt inhibitors, Apc tumors displayed significantly lower *Frzb* and *Sox17* and higher *Wif1* mRNA levels than did healthy Apc mucosae (Figure 4B). Increased *Wif1* levels had already been shown in lesions of Apc^+/min^ mice [26]. Of note, the expression of the Wnt inhibitors was significantly reduced in the normal mucosae of *vil*-TRα1/Apc compared with the normal mucosae of the Apc mice; expression differences were even more pronounced when comparing *vil*-TRα1/Apc tumors to normal *vil*-TRα1/Apc mucosae and *vil*-TRα1/Apc tumors to Apc tumors (Figure 4B). Finally, contrariwise to Apc and *vil*-TRα1/Apc, none of the inhibitors were reduced in tumors from TRα^**0/0**^/Apc mice compared with the healthy mucosae (Supplementary Figure 7A). In addition, their expression was overall increased in both normal mucosae and tumors compared with those of Apc or *vil*-TRα1/Apc mice (Supplementary Figure 7B), except for *Wif1* which showed similar levels in tumors from Apc and TRα^**0/0**^/Apc tumors (Supplementary Figure 7B).

**Figure 5:**
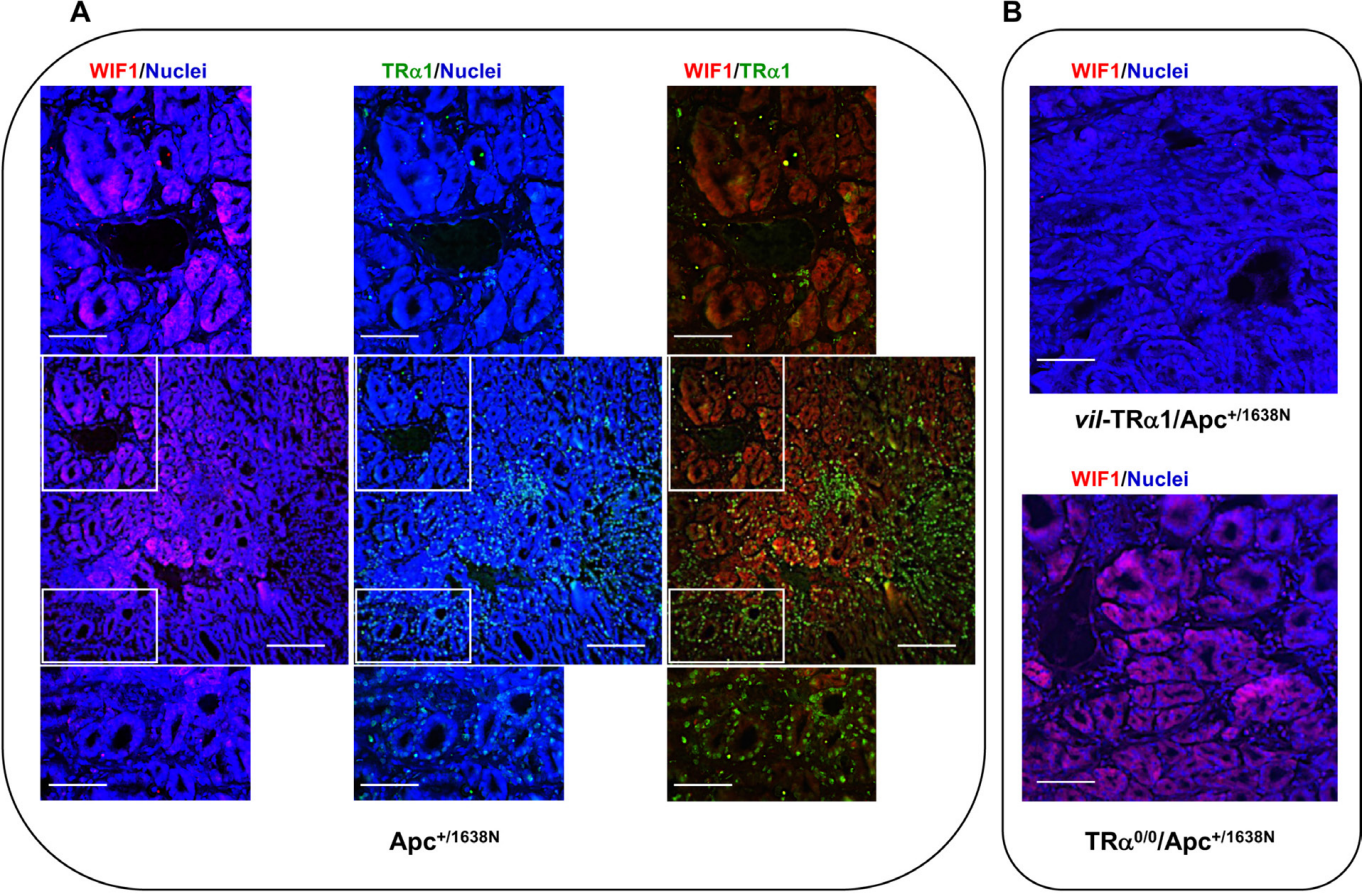
Analysis of TRα1 and WIF1 expression domains Immunolabeling of intestinal tumor sections from Apc (**A**) and *vil*-TRα1/Apc or TRα^0/0/^Apc mice (**B**). The images, which are representative of at least three tumors per genotype, show nuclear staining (blue), protein-specific staining (red or green) and merged images, as indicated. In tumors from Apc mice, TRα1 is heterogeneously expressed, and WIF1 is essentially expressed in areas showing weak TRα1 staining. Bars: A, low magnifications 10 µm, high magnifications 2.5 µm; B, 5 µm.

FRZB and SOX17 proteins were not detected by immunofluorescence or immunoblot in animals of different genotypes (not shown), probably because of their low levels. However, immunostaining of Apc tumor sections revealed the heterogeneous expression of both TRα1 and WIF1 proteins (Figure 5A). Moreover, WIF1 displayed a stronger expression in regions harboring weaker TRα1 staining (Figure 5A). Finally, consistent with mRNA expression levels, the WIF1 protein was not detectable in *vil*-TRα1/Apc tumor sections but resulted more widely expressed in TRα^**0/0**^/Apc tumors (Figure 5B).

**Figure 6:**
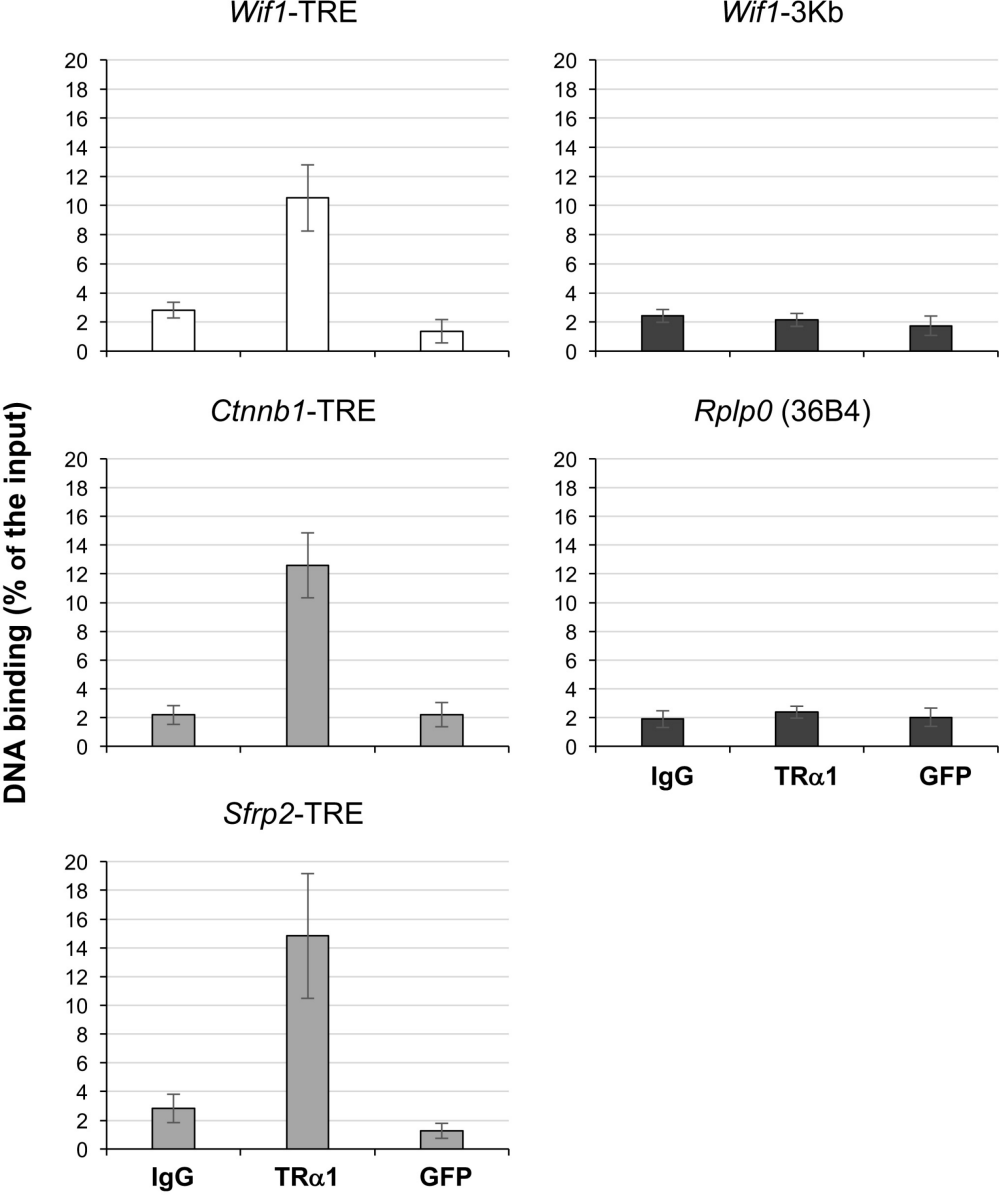
Chromatin occupancy of TRα1 on the *Wif1*-TRE ChIP analysis was performed with chromatin isolated from freshly prepared intestinal epithelial cells from WT mice and immunoprecipitated with anti-TRα1, anti-GFP or rabbit IgG (negative control). qPCR was performed using specific primers covering the *Wif1*-TRE and the *Sfrp2*-TRE or the *Ctnnb1*-TRE (positive controls). Non-specific enrichment of TRα1 was evaluated on a region of the *Wif1* gene located 3 Kb upstream the TRE (*Wif1*-3Kb) and on the *Rplp0* (36B4) promoter (negative controls); the *Ppia* gene was used as an internal control. Histograms represent the mean ± SD, *N =* 3, of the specific DNA enrichment in each sample immunoprecipitated with the indicated antibody and are expressed as percentages of the starting inputs.

Taken together, these results support the model in which TRα1 enhances Wnt signaling at least in part by down-regulating the expression of Wnt antagonists *in vivo*. To decipher the mechanism by which TRα1 is acting on their expression, we investigated the presence of a TRE in the promoter of the Wnt antagonists in order to elucidate whether TRα1 may bind and eventually regulate their transcription. Using an *in silico* approach (http://www.nubiscan.unibas.ch/), we excluded the presence of putative TREs in the *Frzb* and *Sox17* genes, while we located a putative TRE in the promoter of *Wif1*, organized as a canonical DR4 repeat [9], at the position -3555 from the transcription start site (Supplementary Figure 8A and 8B). To check whether TRα1 binds the *Wif1*-TRE *in vivo*, we used a chromatin immunoprecipitation (ChIP) approach. The ChIP assay was performed on fresh epithelial preparations from WT mouse intestine using anti-TRα1, anti-GFP or rabbit IgG (negative controls). As shown in Figure 6 and Supplementary Figure 9, TRα1, but not GFP, bound to the *Wif1* promoter region containing the *Wif1*-TRE site. No binding was observed upstream (up to 3 Kb) of the *Wif1*-TRE site. The percentage of TRα1 binding *in vivo* was similar to that previously described for *Sfrp2*-TRE [27] or *Ctnnb1*-TRE [28] (Figure 6); no specific binding was detected at the *Rplp0* (36B4) promoter (Figure 6).

### The inverse correlation between TRα1 and the Wnt antagonists is also present in CRC patients

**Figure 7:**
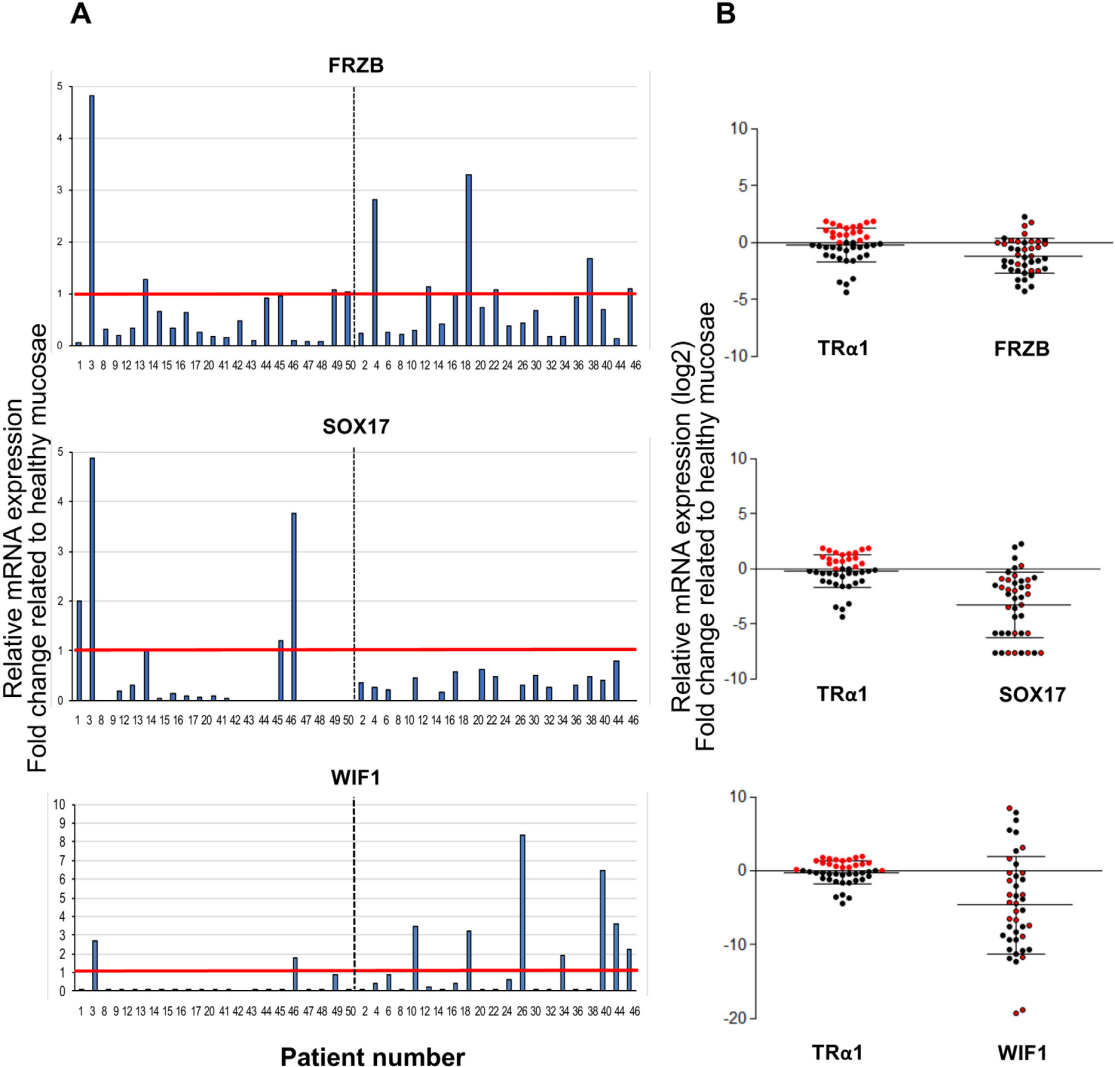
Correlation between TRα1 and Wnt antagonists in human colorectal cancer samples (**A**) Analysis of *FRZB*, *SOX17* and *WIF1* mRNA expression in cohorts of tumors. Histograms display the mRNA expression in each tumor represented as the fold change relative to the expression in the healthy mucosa of the same patient (expression value in healthy mucosa = 1). The red line delineates high- or unchanged/low-mRNA-expressing tumors; the dotted black line distinguishes the two cohorts analyzed. (**B**) Relative mRNA expression of TRα1 and *FRZB* (upper panel), *SOX17* (middle panel) and *WIF1* (lower panel) in each tumor, expressed as fold change relative to the healthy mucosa of the same patient. The thick black lanes indicate the means. Red and black dots show expression values of high and unchanged/low TRα1 expressing-tumors, respectively. *FRZB/SOX17/WIF1* mRNA expression are represented by red and black circles, corresponding respectively to high- or low-TRα1-expressing tumors. Note that most of the red circles are located well above the black dotted line, which defines tumors with unchanged expression levels.

To unveil the relevance of our observations in murine tumor models, we once again examined human CRC cohorts. First, analysis of the TCGA database revealed that *FRZB*, *SOX17* and *WIF1* display lower expression levels in tumors than in normal tissues (Supplementary Figure 10). Next, we verified whether the expression of TRα1 and Wnt antagonists also displayed an inverted correlation in CRC patients. As observed in our murine models, *FRZB*, *SOX17* and *WIF1* mRNA expressions were lower in tumors with respect to healthy mucosae. Specifically, their expression was decreased in 67, 88, and 88% of tumors, respectively (Figure 7A). To better define this inverted correlation, we compared the levels of TRα1 and Wnt inhibitors in each tumor, with each expression being represented as a fold change relative to the respective healthy counterpart from the same patient (Figure 7B). As underlined above, we could distinguish tumors expressing high or unchanged/low levels of TRα1 (red dots, TRα1-high; black dots, TRα1-unchanged/low) within the cohorts. When examining *FRZB*, *SOX17* and *WIF1* expression in the two TRα1 groups, it was clear that low Wnt-inhibitor-expressing tumors (red circles) were essentially those expressing higher levels of TRα1.

Altogether, these results indicate that an inverse correlation exists between TRα1 and *Frzb/FRZB*, *Sox17/SOX17* and *Wif1/WIF1* in tumors concomitantly to a direct correlation between TRα1 and Wnt activity.

## DISCUSSION

Thyroid hormones have been implicated in diseases other than hyper- and hypothyroidism. Several lines of evidence, in fact, suggest that TH and TR receptors may have tumor-promoting or tumor-inhibiting effects. For instance, an increased risk of colon, lung, prostate, and breast cancer with increased TH has been demonstrated in epidemiological studies, even suggesting a TH dose-dependent effect on cancer occurrence [8]. Concerning the TRs, it has been shown that their mutation [29–31] or aberrant expression [29–33] is associated with gastrointestinal tumors. In particular, the *THRB* gene is frequently methylated, and its expression is strongly decreased, in colon cancer [34]. Our data from animal models strongly suggested that the pro-proliferative action of THs and TRα1 on crypt cells [27, 28] plays a major role in tumor development, and further analyses definitively demonstrated that the TRα1 receptor is capable of promoting tumor initiation and progression through an oncogenic synergism with the Wnt pathway [12]. We show here that this synergism affects multiple signaling pathways, tumor suppressors and oncogenes. Importantly, we demonstrate for the first time that TRα1 is up-regulated in human colon cancers and implicated in Wnt activity, thus validating the relevance of our fundamental studies. It is worth noting that the pertinence of murine models of intestinal cancer to human CRC is frequently debated. However, despite the fact that tumors in animal models frequently form in the small intestine, while their distribution in patients is in the colon-rectum, most of their molecular characteristics are similar, independently of their regional origin [35–37]. The *vil*-TRα1/Apc mice, in particular, develop tumors in both the small intestine and colon [12], providing a further proof of their relevance for human CRCs. This point is further reinforced by the current study, as the comparison between human and murine tumors displaying up-regulated TRα1 expression showed similar molecular characteristics when analyzed by *in silico* and by experimental approaches. We can thus conclude that increased TRα1 and its direct correlation with the Wnt pathway is indeed relevant in human CRCs. The importance of this result has been further stressed by the fact that inhibition of TRα1, and thus of Wnt activity, is able to impair the tumor phenotype of colon cancer cells, while TRα1-induced expression has the opposite effect. In addition, loss of TRα1 expression in TRα^0/0^/Apc mice is responsible for delayed tumor development and decreased tumor incidence compared to Apc mutants (Supplementary Table 5), probably because of reduced Wnt activity in a TRα-KO background [28] also dependent upon increased expression of Wnt inhibitors. These findings define new characteristics of CRCs and underline a major role for TRα1 in promoting tumor progression and eventually more aggressive tumor behavior.

Based on a comparative transcriptomic analysis, we established that ectopic expression of TRα1 in an Apc-mutated tumor context results in a very large number of differentially expressed genes, both positively and negatively regulated. Focusing on the Wnt pathway, which was one of the most significantly represented pathways in our data set (*P*-value, 1.9 × 10^–5^), we observed that several inhibitors of Wnt were strongly down-regulated in *vil*-TRα1/Apc tumors. This new result brought an unexpected perspective on the possible mechanisms by which TRα1, through down-regulation of Wnt antagonists, is able to hyperactivate this pathway. In this context, FRZB, SOX17 and WIF1 appeared to be of particular interest. In fact, not only are they *bona fide* inhibitors of Wnt [23–25], but they also showed a strongly reduced expression in the TRα1-transgenic lines used in our study. Moreover, a growing body of evidence links their decreased expression to tumorigenesis or the induction of more aggressive tumors. In fact, *Sox17* functions as an antagonist of the Wnt pathway *via* β-catenin sequestration [24]. In addition, it is frequently methylated in cancers [38, 39], and the inhibition of its expression is associated with cell proliferation [40, 41], tumor progression and poor prognosis in colon cancer patients [42]. Conversely, its re-expression controls processes such as the epithelial-mesenchymal transition and the onset of metastasis [42]. *Frzb*, or *Sfrp3*, belongs to the soluble Frizzled-related protein family, generally considered to be negative modulators of the Wnt pathway and are frequently methylated in advanced stages of colon cancer [25]. *Frzb* knock-down correlates with high Wnt activity and with more aggressive gastric tumors [43]. In addition, a genetic variant harboring a reduced Wnt-antagonist activity is associated with an increased risk of CRC [44]. According to the literature, the *Wif1/WIF1* gene can also be hypermethylated and therefore suppressed in CRC [45]; its repression is associated with increased tumorigenesis or induction of more aggressive tumors [46, 47]. Because of their characteristics, and given that their promoter is silenced by methylation, performing a detailed functional analysis of these genes/proteins in colon adenocarcinoma cell lines or in tumors remains challenging and several of our efforts were unfruitful. We succeeded, however, in showing that at the mRNA level, all three of these Wnt antagonists were inhibited in *vil*-TRα1/Apc normal mucosae and even more so in tumors, while an opposite result was observed in the TRα^**0/0**^ background, strongly suggesting a model of TRα1-induced repression prior to their silencing by methylation in tumors. Their early down-regulation may account for precocious tumor onset.

In our study, *Wif1* behaved differently from the other Wnt antagonists. Increased expression of *Wif1* has been reported in lesions from Apc^+/Min^ mice [26], in agreement with our data on the Apc^+/1638N^ strain, and in lesions of mice treated with carcinogenic agents [48]. These studies suggest that *Wif1* induction represents a response against oncogenic events [26, 48], including strong Wnt signaling, therefore implying that the expression of *Wif1* may be considered a hallmark of a carcinogenetic event prior to the onset of methylation. Interestingly, our results showed that *Wif1* expression, which is induced in Apc and TRα^**0/0**^/Apc tumors, is strongly repressed in *vil*-TRα1/Apc tumors. We postulate that Wnt increases and TRα1 decreases *Wif1* expression, and that the final outcome depends on the relative predominance of each of these actions. Importantly, and supporting this assumption, we showed that *Wif1* regulation by TRα1 might depend on its specific binding through a canonical TRE present on the murine *Wif1* promoter. Given that TRα1 decreases while lack of TRα1 increases *Wif1* expression we hypothesized a possible mechanism involving its TRα1-dependent transcriptional inhibition. Interestingly, we mapped a putative TRE within the promoter of the human *WIF1* gene (Supplementary Figure 11), suggesting that a similar mechanism of transcriptional regulation by TRα1 is present in mouse and in human *Wif1*/*WIF1* promoters, also in accordance with the analysis of the tumor cohorts. It is worth underlining, however, that our molecular analysis only provides the first elements to decipher the possible mechanism by which TRα1 is acting on *Wif1* promoter. An in depth analysis, also taking into account the complexity of studying negatively regulated TR-target genes [49–51], would be necessary to completely unravel this mechanism. Regarding the regulation of *Wif1*/*WIF1* by Wnt, the mechanism remains undefined. Altogether, data from the literature as well as our data do not exclude the existence of other mechanisms involved in the regulation of *Wif1* during the process of tumor initiation and progression. Finally, considering the large list of differentially expressed genes, we can easily envisage that TRα1 boosts tumorigenesis *via* other mechanisms that could involve Wnt or other pathways, which is in line with the Vogelstein model [52].

In conclusion, our study underlined the importance of increased TRα1 expression in intestinal cancer development and progression and described for the first time the relevance of TRα1 expression in human colon cancers. From our analysis it could be proposed that TRα1 together with specific Wnt actors, such as FRZB, SOX17 and WIF1 that are molecular targets of this nuclear receptor, can help define more aggressive tumors and eventually improve the accuracy and precision of personalized medicine approaches to specifically treat TRα1/Wnt-high tumors in patients.

## MATERIALS AND METHODS

### Animals and sample collection

We used WT, TRα^0/0^ [53], *vil*-TRα1 [12], and Apc^+/1638N^ [54] single mutants and *vil*-TRα1/Apc^+/1638N^ [12] or TRα^0/0^/Apc^+/1638N^ (this paper) compound mutant mice.

Animals were housed in the same animal facility and received standard mouse chow and water *ad libitum*. All experiments were performed in compliance with the French and European guidelines for experimental animal studies and approved by the local committees “Comités d’Ethique en Experimentation Animale de l’Université de Lyon” (C2EA15; registration number DR2013-55)”, the Ministère de l’Enseignement Supérieur et de la Recherche, Direction Générale pour la Recherche et l’Innovation, Secrétariat “Autorisation de projet” (agreement 02847.01).

Comparative transcriptomic analysis was performed on tumor samples from *vil*-TRα1/Apc^+/1638N^ and Apc^+/1638N^ mice. Animals were sacrificed, and portions of normal mucosae and tumors were quickly removed under a binocular microscope. Samples were frozen in liquid nitrogen and stored at −80° C for RNA extraction or fixed in 4% paraformaldehyde for histological and immuno-labeling approaches. To assess the levels of circulating thyroid hormones, free T3 and T4 were analyzed by a VIDAS enzyme-linked assay kit (Biomerieux).

### Transcriptome analysis

#### RNA extraction, purification and quality control

Total RNA was extracted from tumors using the RNeasy mini kit and treated with DNAse (Qiagen) according to the manufacturer’s instructions. RNA samples were analyzed quantitatively and qualitatively by NanoDrop ND-2000 UV (Thermo Scientific) and using the BioAnalyzer 2100 (Agilent Technologies). Only high-quality RNA samples were further processed for microarray analysis, with the following criteria: high R.I.N. (RNA Integrity Number), 260/280 nm absorbance >1.8 and 260/230 absorbance >2.

#### Microarray hybridization and data analyses

Labeling, hybridization and detection were carried out by the Biopuces et Sequençage platform at IGBMC (Illkirch, France). Gene expression was determined by hybridization to the Mouse GE 4 × 44 K v2 Microarray Kit (Agilent Technologies). Three independent samples were hybridized for each experimental condition (GEO Accession number: GSE109502).

The raw data after hybridization were imported into GeneSpring GX v 11.5 Software (Agilent Technologies) to be processed, normalized, filtered and analyzed. The procedure includes 4 steps: i) Normalization, ii) Filtering, iii) Identification of differentially expressed genes and iv) Hierarchical clustering.

i) Normalization is the process of adjusting values to improve consistency and reduce bias.

ii) Filtering of genes is an essential step to limit the number of false positives, in order to perform statistics on a robust list of genes. Only the probes present in at least one experimental condition were retained. The criteria for filtering were:

- Uniformity between groups;
- Population non-outlier;
- Unsaturated signal;
- Signal above the background noise.

iii) Statistical tests were applied to identify differentially expressed genes under different experimental conditions. We used the unpaired Student’s *T*-test and one-way ANOVA; in both cases, additional correction procedures for the *P*-values were performed to reduce the number of false positives. For the Student’s *T*-test, the Benjamini-Hochberg correction method was applied, while for one-way ANOVA, Newman-Keuls correction was performed.

iv) Hierarchical clustering identifies group of genes that are logically associated. As a similarity measure, the Pearson centered correlation was used.

#### Ingenuity pathway analysis (IPA)

The association between the genes was further evaluated using Ingenuity Pathway Analysis (Ingenuity^®^ Systems, www.ingenuity.com). Agilent identifiers of the differentially expressed genes and their corresponding expression values (represented as fold change) were loaded into the software and mapped to the corresponding gene objects in the Ingenuity Knowledge Base. The significance (*P*-value) of the associations in functional groups, networks or canonical pathways was calculated using Fisher’s exact test. This estimates the probability that a particular functional classification or category of genes is associated with a particular pattern or cluster of gene expression to a higher level than would be expected by chance. Networks of these selected genes were algorithmically generated based on the relationships between individual genes as derived from literature review and were used to identify the biological functions and/or associated pathological processes. Genes or gene products are represented as nodes in the figures, and biological relationships between nodes are represented as edges (lines). All edges are supported by at least one literature reference from canonical information stored in the Ingenuity Knowledge Base.

### Bioinformatics analysis

#### *THRA* in CRC molecular subtypes

The expression level of the *THRA* gene was analyzed in 566 human colon cancers and 19 non-tumor samples from the GSE39582 dataset [55]. They have been further processed according to the four-subtype consensus molecular classification [13]. ANOVA alone or in conjunction with Tukey’s post hoc test were used to analyze differences between the groups.

#### *THRA* and Wnt signaling

The expression data for 696 colorectal adenocarcinoma (COAD) TCGA samples were downloaded as normalized values from RNA-seq RSEM analyses. The hallmark gene sets from the Molecular Signature Database [56] and the GenePattern 2.0 [57] website were used to score the Wnt signaling per sample (HALLMARK_WNT_BETA_CATENIN_SIGNALING). The Wnt scores and TRα expression were compared using a squared Pearson correlation coefficient (R^2^). All analyses were performed in R [58].

#### Other analyses

Expression analysis of TCGA data (colon adenocarcinoma *vs.* normal colon) was performed using resources present in UALCAN (http://ualcan.path.uab.edu/). Adenocarcinoma samples from stages I–IV were used in our analysis. Level 3 TCGA RNA-seq data corresponding to the primary tumor and normal tissue were used to generate the plots. TPM values were employed for the generation of boxplots and to estimate the significance of differences in gene expression levels between groups. The *T* test was performed using a PERL script with the Comprehensive Perl Archive Network (CPAN) module “Statistics::*T* Test” (http://search.cpan.org/∼yunfang/Statistics-TTest-1.1.0/TTest.pm) [59].

### Human colorectal tumor samples and analyses

One cohort of 59 human primary colorectal tumors (with normal tissue counterparts) was provided by the Tumor Tissue Bank of the Hospices Civils de Lyon (CRB Hospices Civils de Lyon, BB-0033-00046, agreements AC2008-73 and DC2008-72). The tissue bank conforms to French regulations. All patients gave their written informed consent for the use of tissue samples for research purposes. Procedures for collection, storage and release of tissue samples are in accordance with national and international recommendations and a quality management program was developed. To preserve anonymity, a specific ID was attributed to each patient. Fresh tumor and peritumoral colon tissue samples were obtained from surgical resections performed prior to any systemic therapy; tissue samples were immediately snap frozen in liquid nitrogen and used for RNA extraction and RT-qPCR analysis or fixed for immunolabeling. A second cohort from BioChain (colon cancer microarray plate) was included as 24-paired samples (healthy and tumor tissues from the same patient).

### RNA extraction and RTqPCR analysis

Total RNA was extracted using the Nucleospin RNA Kit (Machenery-Nagel). To avoid the presence of contaminating genomic DNA (gDNA), DNase digestion was performed on all preparations. Reverse transcription (RT) was performed with the iScript reverse transcriptase (Bio-Rad) on 1 μg of total RNA according to the manufacturer’s instructions. To further exclude gDNA contamination after RT we conducted a PCR in all preparations to amplify a housekeeping gene (*Hprt* or *PPIB*), the primers for which are located on different exons of the corresponding gene. For qPCR approaches the SYBR qPCR Premix Ex Taq II (Tli RNaseH Plus) (Takara) was used in a CFX connect apparatus (Bio-Rad). In each sample specific mRNA expression was quantified by using the ∆∆Ct method and values normalized against Ppib/PPIB (mouse/human) levels. Primers are listed in Supplementary Table 6.

### Immunofluorescence and immunohistochemistry

Formalin-fixed paraffin-embedded (FFPE) sections (5-mm thickness) were used for indirect immunostaining. Briefly, the sections were deparaffinized in methylcyclohexane, hydrated in ethanol (100%, 90% and 75%), and washed with PBS. The slides were subsequently subjected to antigen retrieval using microwave heating (252 Watts) in 0.01 M citrate buffer, pH 6.0, and incubated for 1 hour at room temperature with blocking buffer (10% normal goat serum, 1% BSA and 0.02% Triton X-100 in PBS). The slides were then incubated with primary antibodies overnight at 4° C followed by incubation with fluorescent secondary antibodies (Alexa Fluor, Life Technologies, 1:1000). All nuclei were counterstained with Hoechst (33342 Molecular Probes^®^). We used the following primary antibodies: anti-TRα1 [27, 28] (dilution 1:50), and anti-WIF1 (Thermo Fisher, dilution 1:250). Fluorescence microscopy and imaging was performed on a Nikon NIE imager right microscope.

Immunohistochemistry on human FFPE samples were performed in the Department of Pathology (Hôpital Herriot, Lyon) and the Pathology Research platform (Leon Bérard Centre, Lyon), according to standard procedures using automated slide stainers.

### Chromatin immunoprecipitation (ChIP) and qPCR analysis

Chromatin immunoprecipitation was performed on collagenase/dispase separated epithelial fragments from adult WT mouse intestine using previously described conditions [27, 28]. We used anti-TRα1 [27, 28] or anti-GFP (Sigma) as negative controls. Specific DNA fragments were analyzed by qPCR using a SYBR qPCR Premix Ex Taq II (Tli RNaseH Plus) (Takara) was used in a CFX connect apparatus (Biorad). qPCR products were then separated and visualized on a 2% agarose gel. We designed primers to amplify a 100 bp sequence containing the putative TRE within the *Wif1* promoter, as well as a 100 bp sequence located 3 Kb downstream of the putative TRE (negative control). In our study we also included primers to amplify genomic regions of already established TRα1 target genes such as *Sfrp2* and *Ctnnb1* [27, 28]. Primer sequences are listed in Supplementary Table 6; the amplified genomic sequence of Ppia was used in all reactions as an internal control.

### Cell proliferation and wound healing assay in Caco2 cells

Studies were performed on the human Caco2 cell line, clone TC7 [60]. ShRNA lentiviral vectors were custom-made Mission-shRNA (derived from pLKO.1-puro, Sigma) based on the TRα1 mRNA sequence provided to the company; Sh sequences targeting TRα1 are listed in Supplementary Table 6. Sh-Scr RNA Control Plasmid (Mission^®^ pLKO.1-puro Non-Target shRNA, Sigma) contains a shRNA insert that does not target any known genes from any species. For overexpression experiments, the TRα1 cDNA was inserted into the Mission^®^ pLKO.1-puro vector (Sigma). The lentiviral particles were generated in our P3 facility and infected cells selected with 0.5 mg/ml puromycin.

Caco2 cells were cultured in DMEM supplemented with 10% heat-inactivated fetal bovine serum (FBS). For cell proliferation studies, 5 × 10^3^ cells/well were seeded onto 96-well plates (Essen Bioscience) and monitored for 70 hours. For wound healing assays 50 × 10^**3**^ cells/well were seeded onto 96-well plates in proliferative (10% FBS) or non-proliferative conditions (in the absence of serum) and the wound was performed the following day using the WoundMaker^™^ (Essen Bioscience). We then monitored the healing over 70 hours. Both assays were performed and analyzed using the Incucyte ZOOM^™^ apparatus and associated software.

### Luciferase reporter assay

Caco2 cells were seeded onto 24-well plates (1 × 10^**6**^ cells/well) in DMEM supplemented with 10% FBS, which contains physiological concentrations of thyroid hormones [61, 62]. For T3 treatments, the cells were maintained in 10% thyroid hormone-depleted FBS [61]. T3 (10^**–6**^ M) or vehicle alone was added to the culture medium 24 hours before the end of the culture. We monitored the Wnt activity using the following vectors: Topflash or Fopflash (Upstate; 200 ng/well); pClneo-β-cateninXL (100 ng/well); EVR2-TCF4E (100 ng/well); pRL-CMV (1 ng/well; Promega). We monitored the T3/TRα1-dependent activity using the following vectors: pGl2-DR4-Luc (200 ng/well); pGS5-TRα1 (100 ng/well) (15); pRL-CMV (1 ng/well; Promega). The plasmids were transfected using the Exgen 500 transfection reagent (Euromedex). Luciferase activity was measured 48 hours post-transfection using the Dual-Luciferase Reporter Assay System (Promega). Data-graphs represent the normalization of beetle-luciferase (Wnt or TR responsive)/renilla-luciferase (not responsive) activities measured in each well, to correct for eventual differences in transfection efficiency from well to well.

### Immunoblotting

Protein samples from Caco2 cells (50 µg per lane) were separated by SDS-PAGE and transferred to PVDF membranes 0.2 μm (Amersham). Membranes were blocked with TBS-Tween (Euromedex) supplemented with 5% non-fat milk before incubation with anti-TRα (Abcam, dilution 1: 500) and anti-actin (Sigma, dilution 1:10,000) primary antibodies. This step was followed by an incubation with HRP-conjugated secondary antibodies (Promega). The signal was analyzed using an enzymatic chemiluminescence detection kit (LumiLight, Roche) and image detection was performed using a Chemidoc XRS+ imaging system (Bio-Rad) according to manufacturer’s protocol. All images were processed using the Image J software.

### Anchorage-dependent tumor growth assay

Anchorage-dependent growth was monitored on solid (plastic) surface as described previously [63]. Briefly, 1 × 10^3^ Caco2 TRα1-depleted or TRα1-overexpressing cells were plated onto 6-well dishes and incubated in 5% CO_2_ at 37° C for ∼2 weeks in DMEM 10% FBS-supplemented medium. Colonies were then stained with 0.005% crystal violet for 1 hour. Each experiment was performed in triplicate and images were analyzed by using the Image J software to quantify colony size.

## SUPPLEMENTARY MATERIALS FIGURES AND TABLES







## Abbreviations

IB: immunoblot
IF: immunofluorescence
IHC: immunohistochemistry
PCR: polymerase chain reaction
RT: reverse transcription
