# Supplementary material for: Increased expression of the thyroid hormone nuclear receptor TRα1 characterizes intestinal tumors with high Wnt activity
Source: Oncotarget. 2018 Jul 24;9(57):30979–96. doi: 10.18632/oncotarget.25741 (PMC6089551; doi:10.18632/oncotarget.25741)
Supplement: Supplementary file 1 [file oncotarget-09-30979-s001.pdf]

Increased expression of the thyroid hormone nuclear receptor TRα1 characterizes intestinal tumors with high Wnt activity

SUPPLEMENTARY MATERIALS

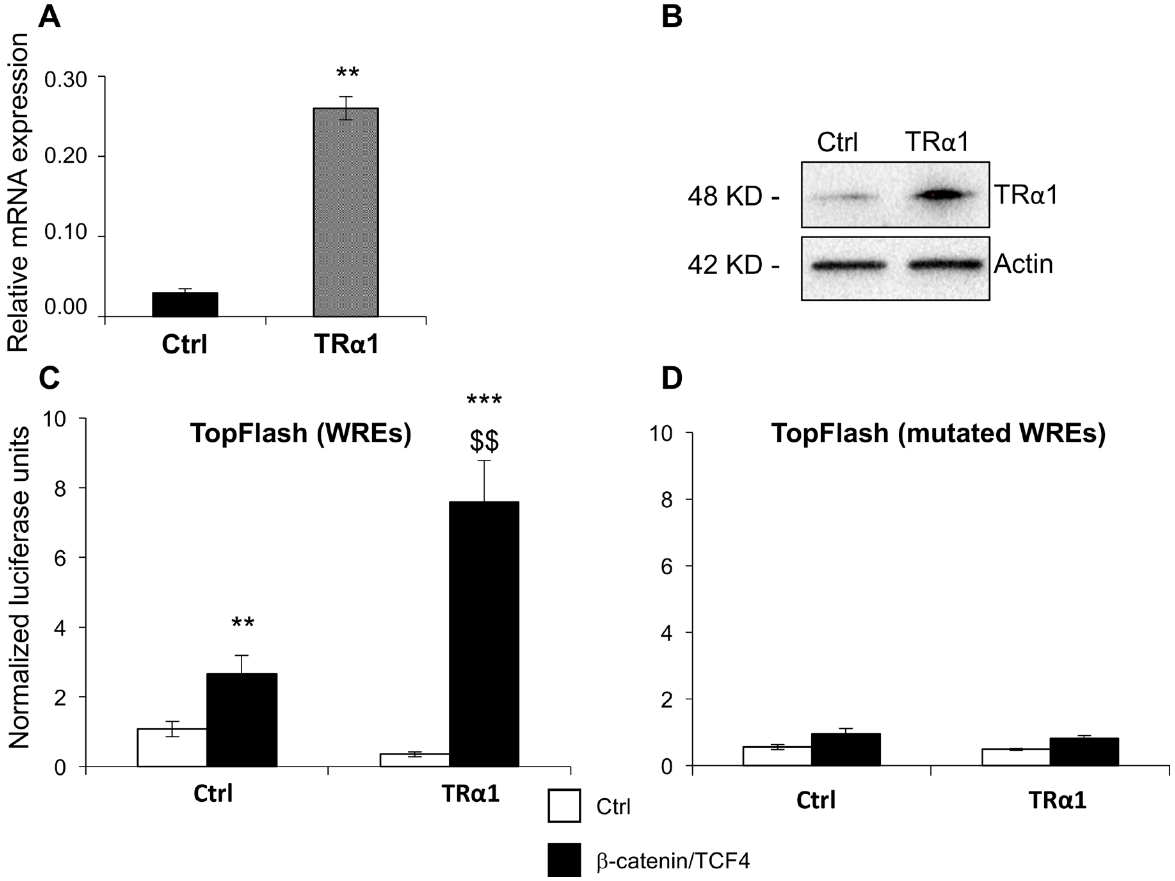

Supplementary Figure 1: Increased expression of TRα1 hyper-activates canonical Wnt/β-catenin signalling pathway. (**A**, **B**) Generation of stably overexpressing TRα1 Caco2 cell line. The overexpression efficiency was assessed by comparing TRα1 mRNA by RTqPCR (A) or protein by IB (B). $^{**}P < 0.01$ compared with control condition (Ctrl) by Student's *T*-test. (**C**, **D**) Increased TRα1 expression potentiates canonical Wnt signalling activity. Bar graphs display quantification of luciferase activity in Caco2 cell lines generated in A after transfection with a TopFlash vector (C) or the negative control FopFlash vector (D) to monitor Wnt activity using the Dual-Luciferase Reporter Assay System. Caco2 cells were maintained in culture medium containing physiological concentrations of T3 in the absence (Ctrl) or in presence of co-transfected β-catenin/TCF4 complex. Histograms represent mean ± SD ($N = 6$) from one out of three independent experiments. $^{**}P < 0.01$ and $^{***}P < 0.001$ compared with the control condition of the same cell line; $^{\$\$}P < 0.01$ compared with the β-catenin/TCF4 condition of the control cells, by Student's *T*-test.

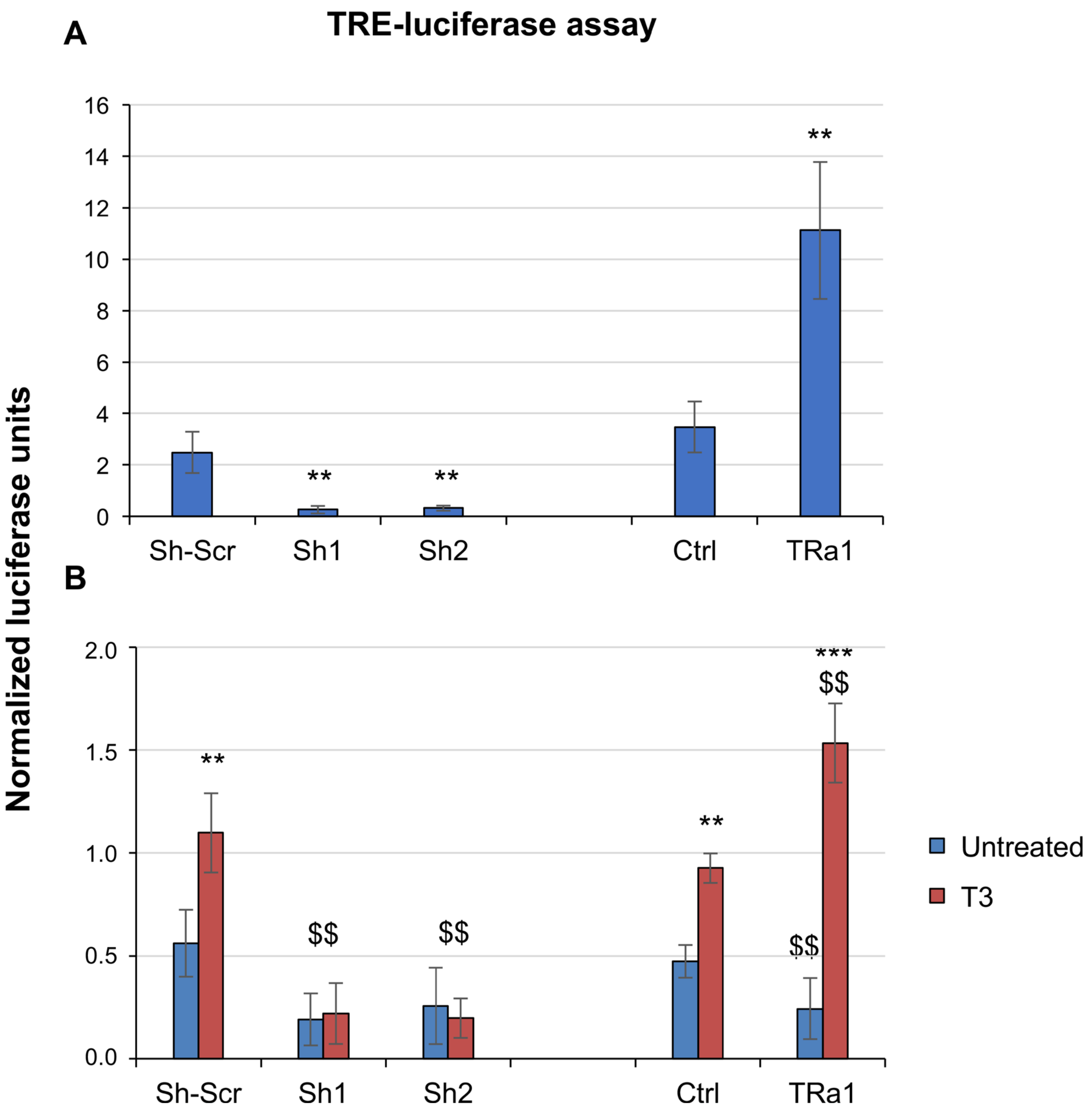

Supplementary Figure 2: Responsiveness of the different Caco2 cell lines to a TRE-luciferase assay *in vitro*. (**A**) The TRE-luciferase reporter was transfected into Caco2 cells maintained in culture medium supplemented with 10% FBS, thus containing physiological concentrations of T3. Histograms represent mean ± SD from two independent experiments, each conducted in six replicates ($n = 6$). $^{**}P < 0.01$ compared with the respective control condition (Sh-Scr or Ctrl) by Student's *t*-test. (**B**) The TRE-luciferase reporter was transfected into Caco2 cells maintained in culture medium supplemented with 10% of T3-depleted FBS in the presence (T3) or absence (untreated) of 10^{-6} M T3, as indicated. Histograms represent mean ± SD from two independent experiments, each conducted in six replicates ($n = 6$). $^{**}P < 0.01$, $^{***}P < 0.001$ compared with the respective untreated condition; $^{\$\$}P < 0.01$ compared with Sh-Scr or Ctrl cell line in the same condition, by Student's *t*-test.

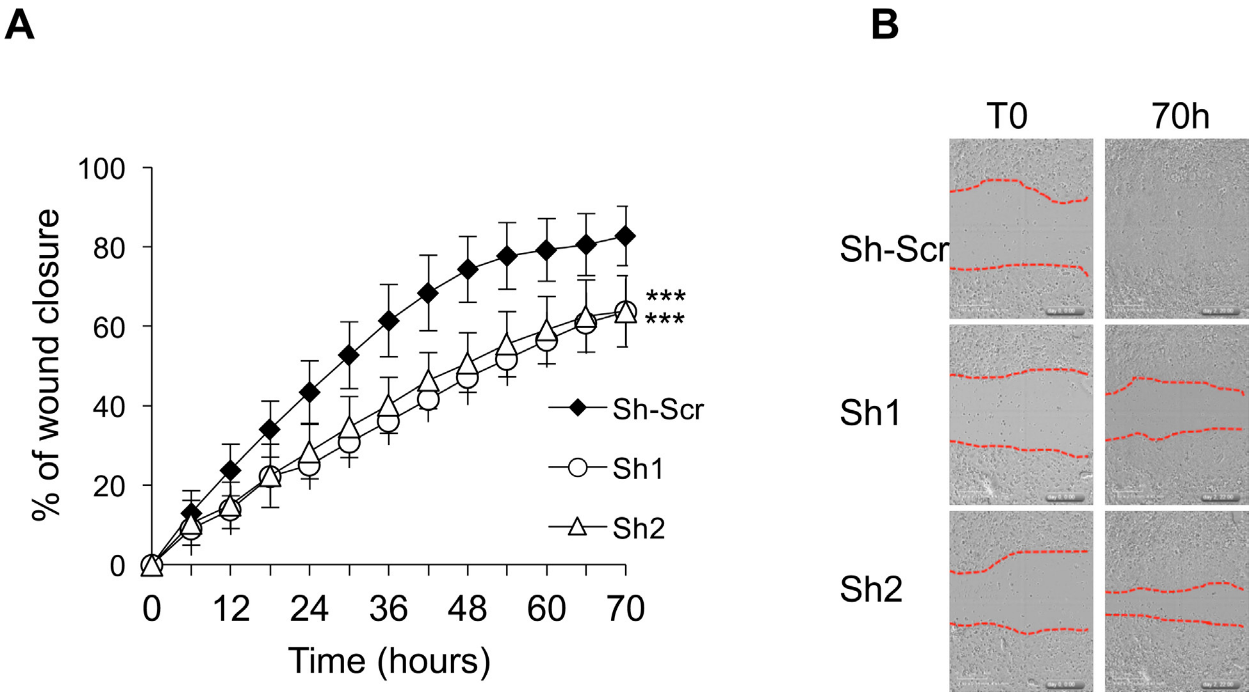

Supplementary Figure 3: Complementary analysis of TRα1-dependent cell migration in colorectal cancer cells. Depletion of TRα1 inhibits 2-D cell migration. Confluent monolayers of Caco2 cells were scratch-wounded and incubated for 70 hr in FBS-deprived non-proliferative condition, and wound closure was monitored and quantified. In panel (**A**) confluence indexes (y-axis) in cell lines are displayed as line graphs. Note that depletion of TRα1 significantly delays wound closure. Histograms represent mean ± SEM ($N = 10$) from one of three independent experiments, each conducted in 10 replicates. Panel (**B**) shows representative pictures of different cell lines taken at T0 or T70. Red dashed lines in B delineate the wounded surface (T0) and the surface still depleted of cells (T70). $^{***}P < 0.001$ and $^{****}P < 0.0001$ by Student's *T*-test. *** in (A) applies to each time-point.

A

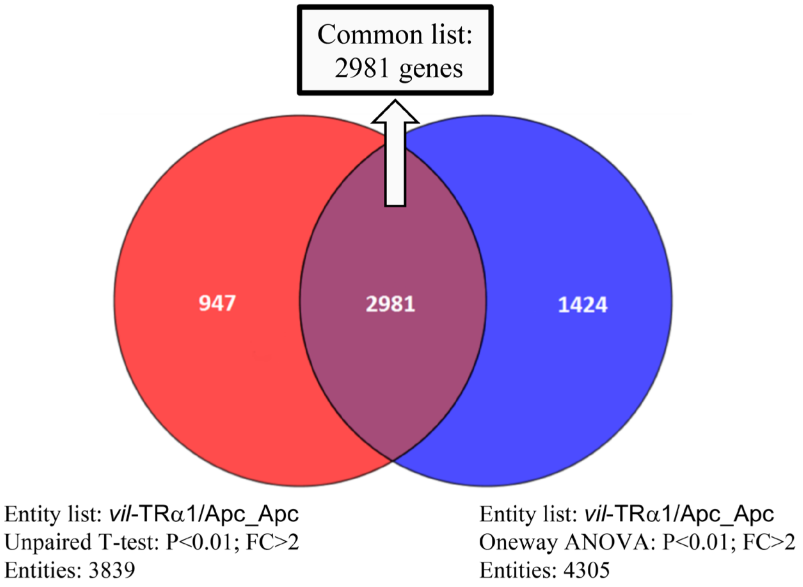

B

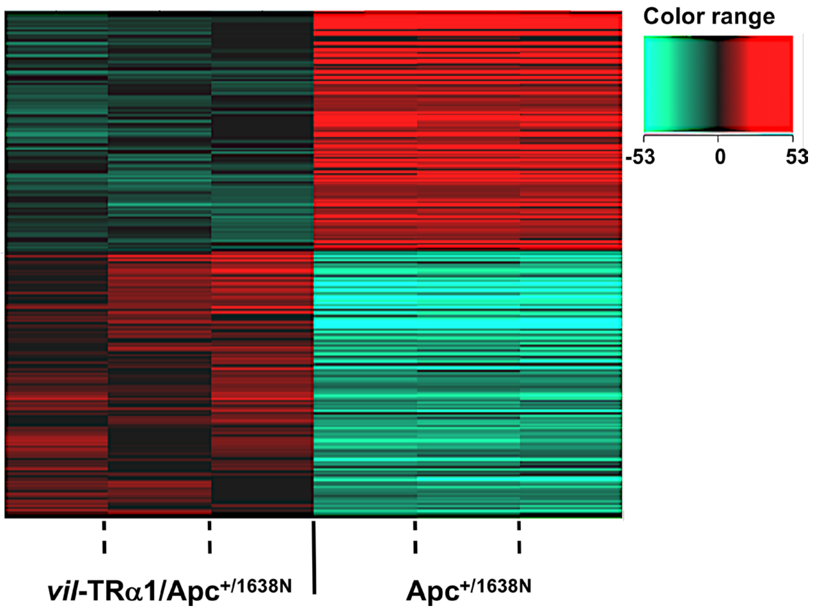

Supplementary Figure 4: Generation of a list of differentially expressed genes and their hierarchical clustering. (**A**) Venn Diagram representing the 3839 differentially expressed genes obtained by performing the *T*-test (left), the 4305 differentially expressed genes obtained with the ANOVA test (right) and the 2981 common genes. For further studies we focused specifically on the common list. (**B**) Hierarchical clustering enabled to observe the relations between the tumors of different genotype and to visualize the differentially expressed genes. In this clustering, the 2981 transcripts were grouped in two dendrograms, each of which represents a genotype. Each line is a probe and each column is an RNA sample hybridized on an array slide. Expression signal intensities are shown in red and green, indicating high and low expression levels respectively. Dotted lines delineate each individual sample; adenocarcinoma samples of different genotype are separated by the thick line.

A

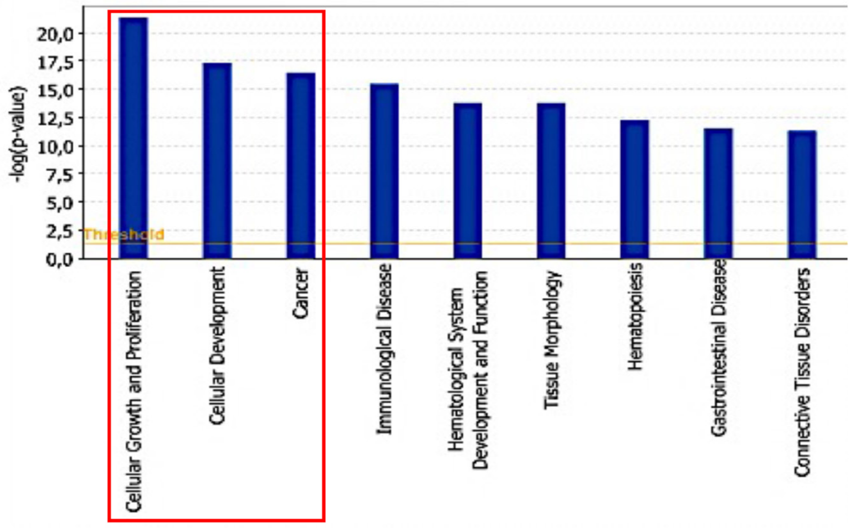

Category	Functions Annotation	P-value	Genes
Cellular Growth and Proliferation	Proliferation of cells	4.35E-22	423
Cellular Development	Differentiation	4.51E-18	316
Cancer	Tumorigenesis	3.36E-17	558

B

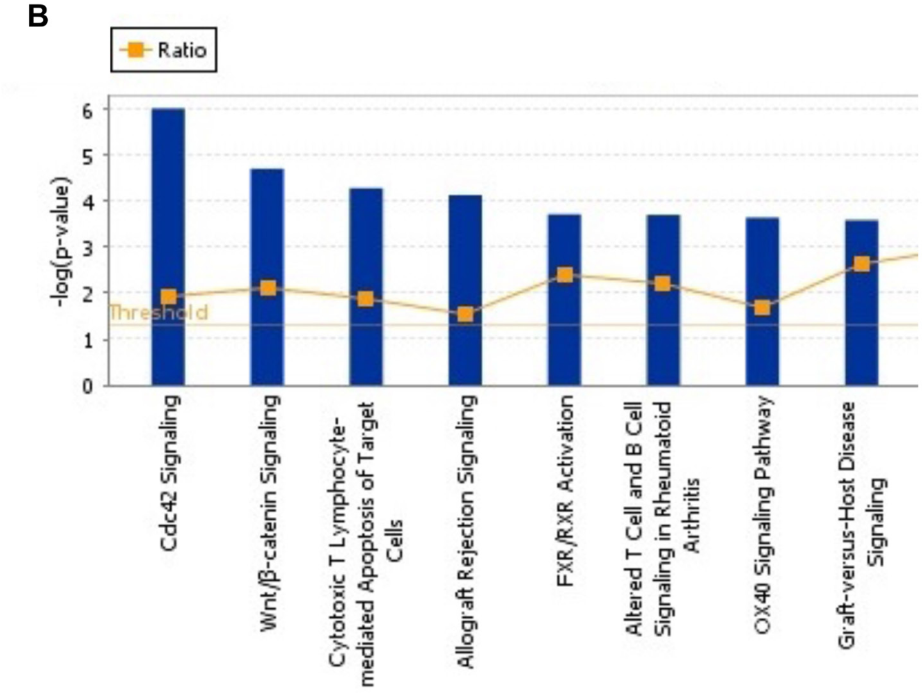

Supplementary Figure 5: Gene ontology analysis for significantly changed transcripts by Ingenuity Pathway Analysis. (**A**) Bar chart illustrating Biological Functions associated with the differentially expressed genes. Table summarizing the top 3 functions, their statistical significance and the number of genes present in each category. (**B**) Bar chart of the most significant canonical pathways associated with the differentially expressed genes.

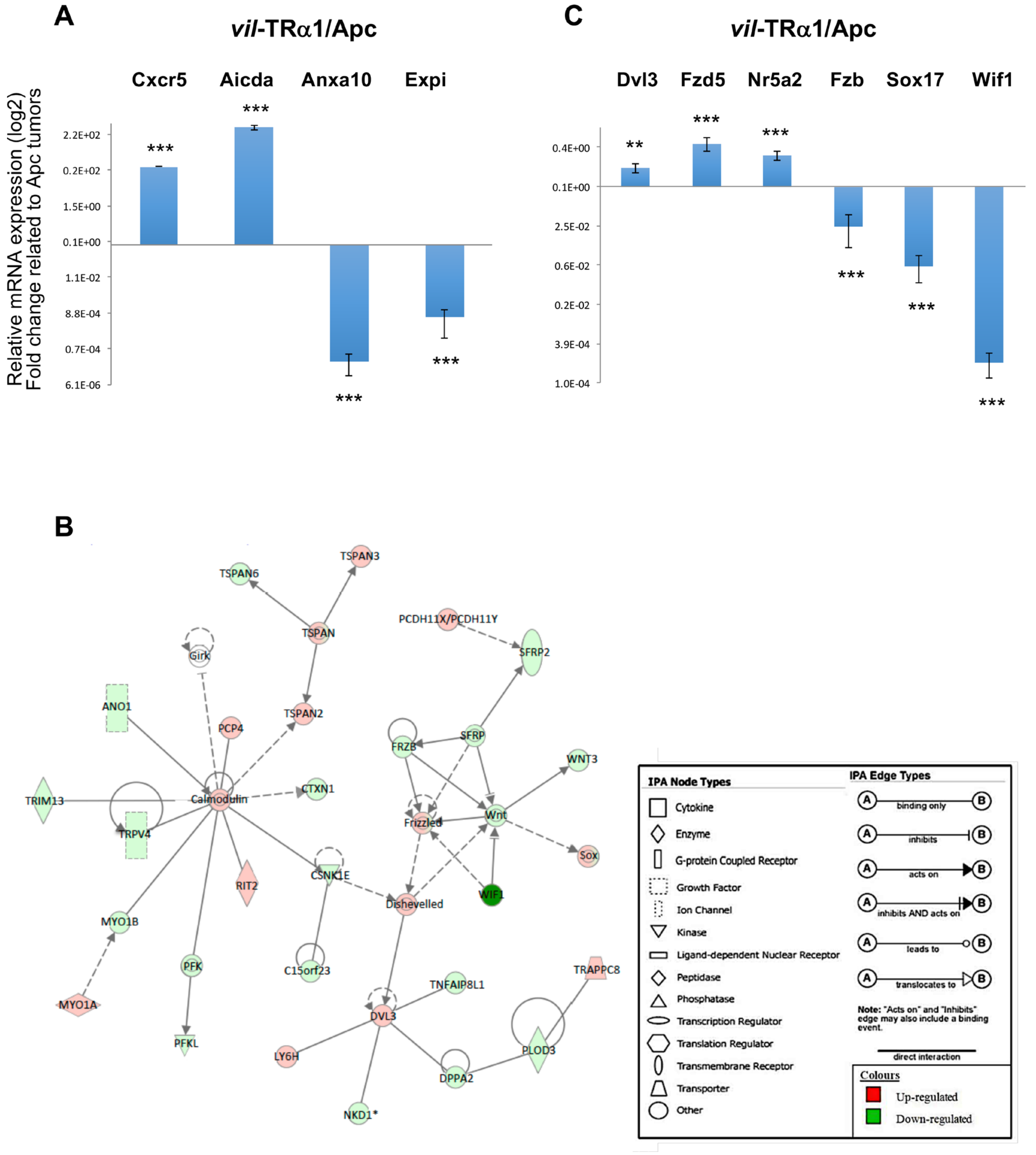

Supplementary Figure 6: Differentially expressed gene validation and modeling. (**A**) Analysis of strongly regulated genes by RTqPCR. Histograms show fold changes in *vil*-TRα1/Apc tumors related to Apc tumors. $^{***}P < 0.001$ compared with Apc tumors by Student's *T*-test. (**B**) Molecular relationships between transcripts in the identified network "Cellular growth, Cell proliferation and Cancer" by Ingenuity Pathway Analysis. Genes or gene products are represented as nodes and connections between genes are supported by information in the Ingenuity Knowledge Base. The color indicates upregulation (red) or downregulation (green). Different shapes of nodes represent different functional classes of gene products as indicated in the legend inset (right panel). (**C**) Analysis of the Wnt agonists and antagonists present within the gene network in B. Histograms show fold changes in *vil*-TRα1/Apc tumors related to Apc tumors. $^{**}P < 0.01$ and $^{***}P < 0.001$ compared with Apc tumors by Student's *T*-test. Note that agonists are up-regulated and the antagonists are down-regulated in TRα1-overexpressing tumors.

A

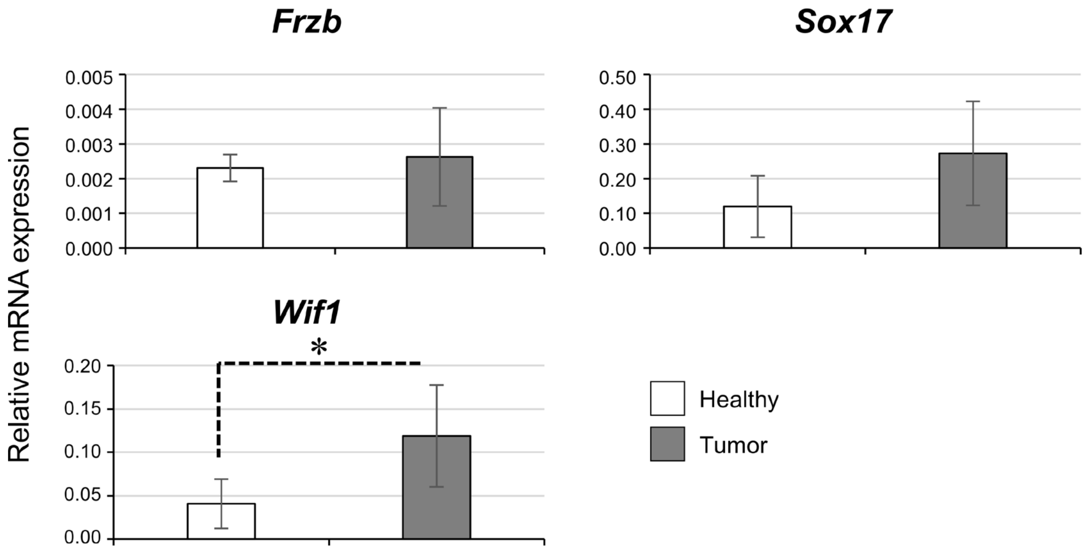

B

	Fold change TRα$^{0/0}$/Apc *vs* Apc		Fold change TRα$^{0/0}$/Apc *vs* *vil*-TRα1/Apc	
	Healthy	Tumor	Healthy	Tumor
Frzb	0.8^{NS}	1.7*	1.5*	7.6**
Sox17	1.6^{NS}	5.6*	3.4*	92.7**
Wif1	1.5^{NS}	0.8^{NS}	5.1**	372***

Supplementary Figure 7: Expression of Wnt inhibitors in TRα0/0/Apc animals. (**A**) Analysis of Frzb, Sox17 and Wif1 mRNA expression in healthy mucosae and tumors from TRα0/0/Apc animals. Histograms represent mean ± SD. $^{*}P < 0.05$ by Student's *t*-test ($N = 6/8$). (**B**) Comparative study of the inhibitor's mRNA expression in healthy mucosae or tumors from TRα0/0/Apc animals and those from Apc or vil-TRα1/Apc mice. $^{*}P < 0.05$, $^{**}P < 0.01$, $^{***}P < 0.001$, by Student's *T*-test. NS: not significant.

A

AATGCTGTAATGCTGTGAAAAAAATGCTCAGAATTTTAGCTGCCAGATTTCCTCTTTGAACTTCTGTCAAAAGTTGCTGTACTCTGCTTTATATCAGGGTTGTGAGGC
GCTTGTCGTCTTAGCGAGCCTGGTGCTCTGAGGGGCTCAAGTTCTGTTTTTTTCTTGCTTT**AGGTCA**GTGC**TGGGCA**TTCAGTAAATCTTATGGAATGAGTGAA
TAAATTAATAGACAGATGAATGAGGCTAGAAACACAGTGGTCTGGCTGAATCACACAAGGGAAGGAAGCATTCTGGGTTAGACAATGTATGGGAGAATTCTCAAAGCA
AACACAACAACTCCTAACACACTGTAGCCAACATCATGCATGCGACTATATTTTATTAGAAAATTTTAAAAGCATACCTATTCTAAATGAATAAACTTATTTAAAAAG
GGGGGATAAAATTTCAGTTCTTGGATAGATTTCCTATAGAGATACGCTCTATTACTGTTAAGTATCTGACCTGCCTACACAAAGCATGTTTCTTCCCCCTTAAAACCC
AAGTTATTCAAACACAGACTTCTTTAAGACAGGATTTTGCTTTGTAGCTCAACTAGCCATTTAGGCGTCAGAGGACTGAAATTACAAGATGTCACGTGCCACCACAGC
TGGCTTAAATTTAGGTGTGAGTGAGTGTGTGTGTGTGTGACTGCATATGTGAGTGTGTATGAGTGACTGTATATGTGAATGTATGTGAGTGTATGTGAATGTGTGTGT
GTGAGTGTGTATGTGAGTGAATGTGTATGGGTATGTTGGGTGTGGGGGAGTGTGTATTGTAAGCATGTTTGTGTGAGATTTTGTGTTATGAGTGTGTGAGGTATGTAT
ATATGTGTTTGAGTGTGTGAGTGTTTGTGTGTGTTGTGTGTGAGTGTGTATAAGTGTGTGTTGTGAGCATGTATATGTGTGAGTGTGTGTGCATGCGTATATGTTGTG
TGTGAGTGTGCATGTGTGTGTATATGGGTGTGTGTTGTGAGCATGTGCACGTGTGTGTGTTTGTGTGCACACATGTGCATGTGGTGCACACAGGACAGAGGGGACATC
AGCTGTCTTGCTGAATCGCTTTTCAATTACTCCCTGAGGCAGGAGTTCTCACTAACCCAGAGCTAGGCTAGCCACCAGTAAGTCCCAGGCATCCTCCTGTTGCAGCGC
CTCAGCACTAGGGTTACAGGAGCACAATACCTCGGACAGCTTTTTATTTGTTTGCCAGGGATATGAACTCTGGTCCTCGCGCCTGATCAGTAAGGGTAAAGACTCTCA
CCGGCTTAGCTATCTCTCTAGTACCAATAATTTTTAAATAACGTTTTATGTATCTCTAAAAATACAGTATGTTTCCTGTTGGAGAAAAGTGAACAGAAAAATGTAGTT
GGTAGTATTGGTGGTGCTGTTGCTGTGGCCACTGATGATGGTGATGATAATGATGATATAACACAGGCTACTTTCTCTGTAGTCTAAGCTGGACTTGAATTCACTAGT
AGCCTCGGCTGGCCTCATTTTCACTGAAACCCTCCTGCCTTAGCCTCTGAGTGCGGGGATAACAGGCATTTGCCACCACACCTAACAAAACAATTGTCCAGAATTGGG
GAAATTTCTTGATAGCAGGATAAATTGCTGTCTCAACATCAAAGACGACTGCTACGGAAAATCCTCACTTGGAGGAAGCTGTATTATGATAGAATTTGTTCCCCTTTC
AGTTCTCAGCTGGCGTTTGACAAGAACAACCTGTAGAGGGCGTCCTTACACCAAGAGCCATGGAGGTCTGAGCAATACTACGGAAGAAAAACCTTCAACGAAAACCAC
ATTCCAAGTCAAAAAGAGTCTATTGTGTTGCGAAATTATTACCTGTAACTGAATGACACTGTTTTTACAATATTAAAGGGTTATAAGTTTGCCTAATATAGTAAAACA
TTATATAAATTAATATTCACTGCAACAATTAATTAATAAAATAGAATGTTGGGAAAAGATCCTTTTTTGTTTGTTTTTTGTTGTTTTTGTTTTTGTTTTTTTGAGACA
GAGTCCCAGGTAGCCCAGGCTGGCTTCAAACTCCATATGTCGATTAGTATTAACAATGAGCTCCTGATGCCTCTGCCTCTACTTCTTTAGCCCCCAGATTACAGCCTG
TGCTACCACAACCAGTTTAAATGTTTTGTTTTAAAGAAAGAGTAGATGTTAAGCTTTTTGAGGCAAACCGTCTTAATTGTGATTTGCAAGAAGGGGCTTTGAAGTTA
GAGCGATCGTGATTTGAATTCCTACTCTTGTCACTCACCAGCTGAATATCTGCGGTCAATTTACTAGACTTTTAAAGCTTTGGTTCCCCATCAGTAAAATGAGAGTAG
TAACAGTAGCTGCCTGAGAAGAATTTTTAAAATGAAGCTTCATGGAGTTCACAGTCACAATGGACAGTCTTCGTGTCTAACTCCCAAACACAATACCTTCCTACCAAT
CTGTTAGATACCTACACAGATTTCACAGTTTTAACACATGTTTCTTCTTCTTCTTCTTCTTCTTCTTCTTCTTCTTCTTCTTCTTCTTCTTCTTCTTCTTCTTCT
TCCTCTTCTTCTCCTCTTCCTCCTCCTCCTCCTCCTCCTCCTCCTCCTCCTCCTCCTTCTCTCCTCCTCCTCCTCCCTTCCCATTCCTCCTTCTTTTAAAAAAATATG
CTTGTGACCTAACATTTGTTGAACATGTACGCGTCCAAGTCCGGCTTCTATTCTGTAGTTTCTGTTTTTGCCATCCCCTGCTGAGAGACCCACCGCTCTACAAAGGAC
AGAAGGGAACACAGCCCAGCGCCAGAGTCTAGATCCTTGGGGGGCATTTGCTAGAAAGCCACTGGGTGCAAAAAAAAAAAAAAAAAAAAAAAAGTAAACAAAGCTTTTCC
GGGGTCTGAAAACTTTCTGTGTTTTTGAAAAACCATTTGTTTCTGCTTTCTCTGTACGTTCAAGGAGGCAGGCAAAAAAGCCTTCCGTGCAGAGGTAATGCTGCAAGC
CGCCGCTAGCTAAGTGCTCTCCCACCAGACCCTCAGTCTTACCCTCAAGCGCCTAAGATTTCAGAGACTGGGCGTGAGCCTCCTAATGTTTTCCCCCAAATCCAATTT
TAAGGGGAGTCCTACCGTCGTAGAGCAGGGATGGGGGGAAAGGGATTACAGCTATCATCGAGGTCCTAGGGAACTATTCCGGGTGCAACTGGGTTATCAAGAAGACAA
ACCACGCCACCTGTCGGGTGTCTGTCCAGTGCAGAAATAAATGAACGTCCTGCGAGGACCAGCGCCTGCAGTAGCCCGACTAGCCTGTGGAGTCTCAGTATTTAACTT
CCTAGGAGAGAGAGAGCGAGAGCGCAAACTGTCAGCTAGAGAAGTCAGAGTTTGCAGACAGGCACACAGAGATGCAGTCTGAAATGTAGTTCGAAGAGAAAGGGTGGG
CTGGCTATGTGCTTTCCGTTAGCTCCACTGTTAAACGGAATTTATCTTTGACCACGAATCGAGCCCAGATTTTTTTGTTTGTTTGTTTATAGCACCCCGGCCATCCAC
ACGTGTCTCTTCGGGCGGCAGGAGGCCTGAGTGATGACCCAGAAGTCCCTTTGGGTGTGTTTCTTGGTTGTGTCCCCGTTTTCATTCACCTGGCGCAGGCGAGCGGGG
TCTACCGGGTACCGCGGCCCCGCCCCCGCGGCTCCGCAGCGCTCCCTAGTGGCGACCGCCTGCACACCACCGGCCGCTCTCAGTGCTCGCCTTTTGCCAGCAGTTCCC
GCGGTTCCTCCCTCTCGAGTGTGCTCCTCTAGCTGCTGGCGCTGTGGGACGTGCTGGCATTGCCAGCTTTGCCAGCATCGTGTCTCTGCGTCCCCCTCTCCCTCCAGC
CCCCACCTCTTTCTACGCGGCCGCGCATCCACTCCCTCCTTGCTCCCTCCTCTCCAGCACTCCCAGGCTCACTCGCAACGGAGACTGGAGACTTGCACGCCCAGTCGT
TTAAGTAGGAACAGCTCCAGCCCCGCCAGCTGCAGCCAAGGCGAGAACTTCACAAGCAGCACAGGTTGGGTCGCTGCGGCAGGAGTTGCACCACCAGCGAGAAGGTCC
TGAGCACC**ATG**GCTCGGAGAAGAGCCTTCCCTGCTTTCGCGCTCCGGCTCTGGAGCATCCTACCTTGCCTGCTCCTGCTGCGAGCGGATGCAGGGCAGCCACCTGAG
GAGAGCTTGTACCTGTGGATCGACGCCCATCAGGCTAGAGTGCTCATAG

B

Species	TRE	Position (strand)	Score	P-value	Site sequence
Mus musculus	DR4	-3555(+)	0.756366	0.0237602	AGGTCAgtgcTGGGCA

Supplementary Figure 8: Localization of the putative TRα1-binding site on the murine Wif1 promoter. (**A**) A 5 Kb region upstream of the Wif1 start site was analyzed using the NUBISCAN software, revealing one putative binding sites. The sequence highlighted in bold (yellow background) shows the arrangement of the two half-sites as a typical DR4. The first exon is in red, and the first coding ATG is in bold in a yellow background. (**B**) Results obtained by NUBISCAN summarizing the score, the P-value and the sequence of the putative DR4.

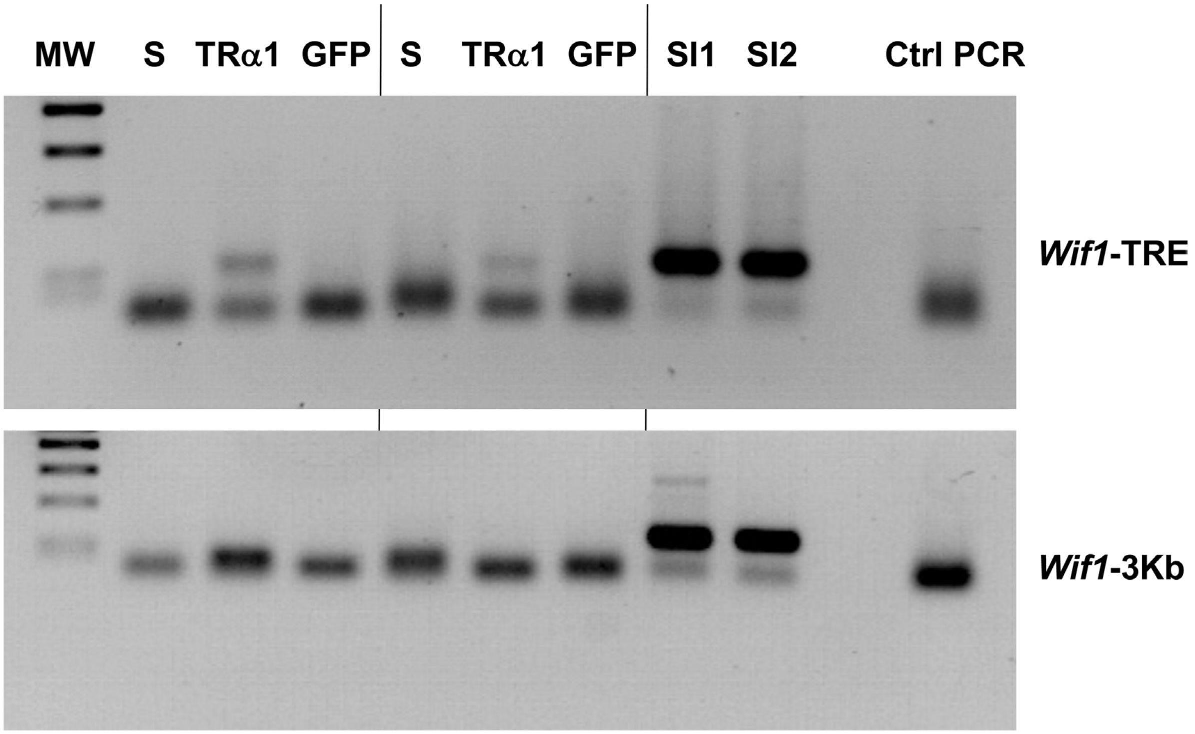

Supplementary Figure 9: Molecular analysis of the Thyroid hormone Responsive Element present in the Wif1 gene, by in vivo chromatin immunoprecipitation. Study by qPCR of the DNA purified from the different samples before and after ChIP visualized on agarose gel. The picture is representative of two independent experiments. Indicated on the right part of each panel is the fragment of amplified Wif1 gene. S: pre-immune serum; TRα1: anti-TRα1; GFP: anti-GFP; SI: starting input; Ctrl PCR: negative control for PCR mix.

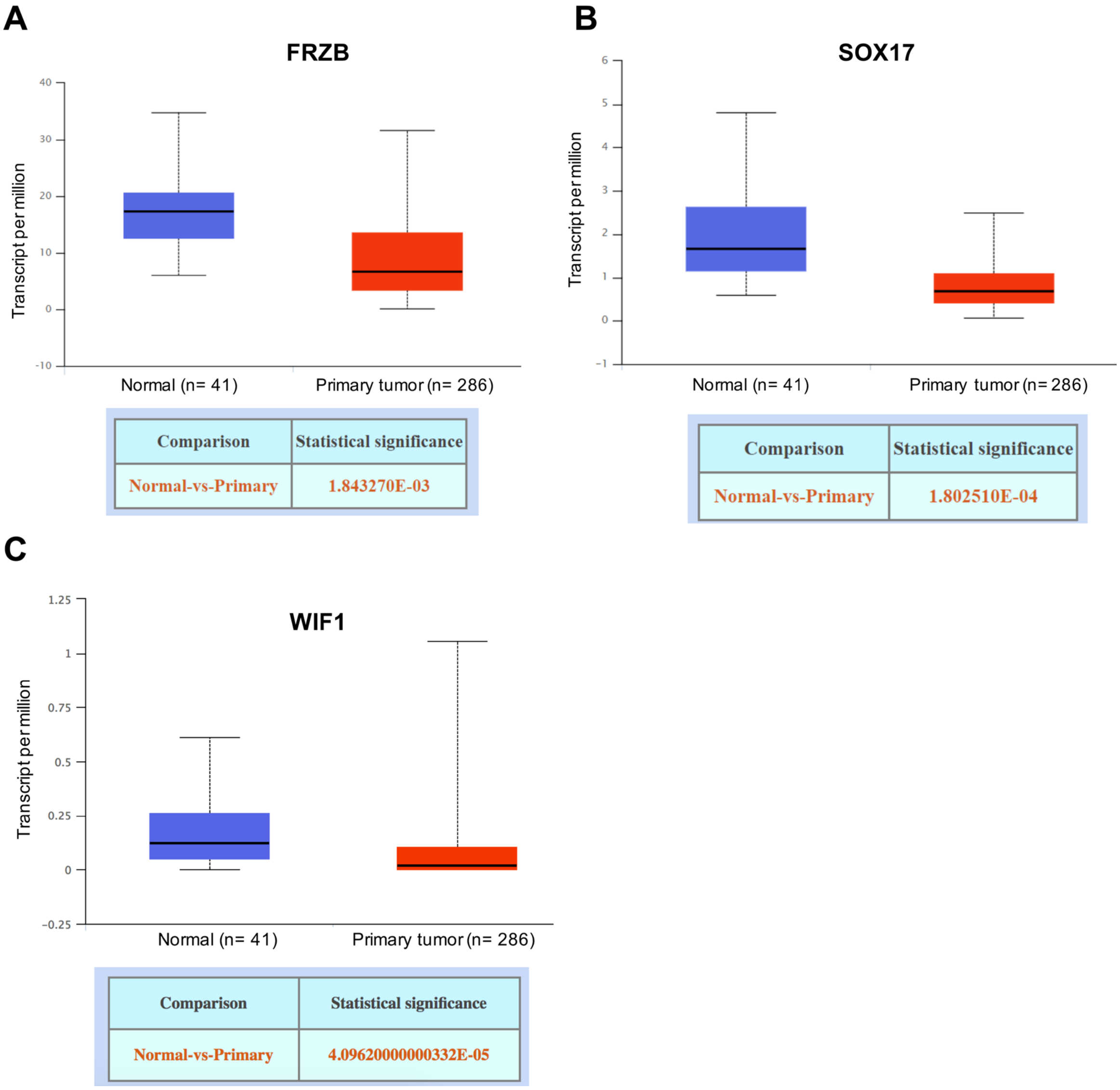

Comparison	Statistical significance
Normal-vs-Primary	1.843270E-03

Comparison	Statistical significance
Normal-vs-Primary	1.802510E-04

Comparison	Statistical significance
Normal-vs-Primary	4.09620000000332E-05

Supplementary Figure 10: Analysis of FRZB, SOX17 and WIF1 mRNA expression in CRC patients. (**A**) FRZB, (**B**) SOX17 and (**C**) WIF1 mRNA expression values in TCGA (The Cancer Genome Atlas) dataset. Boxplots illustrate the distribution of data and the mean (black thick line). Note that all of the Wnt antagonists are significantly down-regulated in CRCs, as indicated.

A

TCTGTTGTTTCTGTTTGTTTTTCCCTCTTGAGGCATCTGCTACTCTATAAAGGACTGATTGAAACACCACGCCCAGCCCCAGACATTTGATCCTTGAGGCATTTGTCA
CAGCTTCGGGTACTAAAGAACAAACACAAGTAAATGAACCTTTTATGAGTCCGAAGGCTAAGTTTTTTAATTAAAAACAAAACAAAACAAAACCCTTTTGCTTCCGTT
TTCTTTGTGCATCCGAGGAATTAGGCAGGAGGCAAAAATCAGTTCTTTCTGCTGGGCCAGGTGGATAGGTTATGCTGCAGCCCACTGCCGGCCAAGCATGCTTCTCAC
AGACGAGGCCAGAGCCTTGGTTTTGCCCCCACGCACCTGTGAATTCGGCAACTTTTAAAAAATTATTTTATTTATTTATTTATATATTTTTTGAGACGGAGTCTCGCT
CTATCGCCCAGGCTGGAGTGCAGTGCCACGATCTTGGCTTAATGCAACCTCCGCCTCCCGGTTCAAGTGATTCTCCTGCCTCAGCCTCCCGGGTAGCTAGGACTACAG
GCCCCCGCCACCATGCCCAGCTAATTTTTGCATTTTTAGTAGAGACGGGGTTTCACCACGCTGGCCAAGCTGGTCTCGAACTCC**TGACCT**CAGG**TGATCC**GCCC
GCCTTGGCCTCCCAAAGTGCAGGGATTACAGGCGTGAGCCATCGCGCCCGGCCGAATTCAGCAACTTTTAAAAAATATCAGCAAACGTGAAGATATCCACGATGTTAG
AGGAGCCCTACCCCGGAGGGTCAGGTACAGCTATCGTCCAGGGCCCAGGGCACTCTTCCGGGCACTGCCGGGTTATCAGGGAGACAGACGGGAATCCCCAAATGCTGG
GTGTCGGGCAAGTACCAGCTGGACGCCCTGCGCCTCGAGCCAAGGCCAGCGCCTGCCATCGGCACCATCGGACAGTCGAGCCTCGAGTTTTAACTGCTTGGGAGCGCG
CAAAGTGCCAGCCTATCGCAGAACGGAGCGCATAGGGTTGGCGGAGAGAGGAATCCTACTGGCTGAAAGGGAGACGAAGGGCAATTTGCGCCTTCAGTGAGCGCCGGA
GGAGGAACAGGAGTCATCACCTCATCATCATCATCATCATCATCATCACCATCACCATCACCATCATCAGCACTCAGTCAAGCCCAGCGTTGTCTGCTCTCCCCATTT
CCCTCCCCCGAAGCCTCCCTTGGCCCGAGGAGGTGGCGAGTGATGTCCCAGGGGTCTCTGAGTGCCCTTCTCCGGGTCCGCCAGCCCTACACGCCCACTTCGCGGGCG
CTCCACTGGGCGCACCGCACTGTGAATGCAGCCTCGGGGGTCCCTCGCGGCCCCGCCCCCGGGGGGGCCCCACAGCGCCCCCAAGTGGCGGCCGCCCAGGCCTCGCGG
GCCCCACTCCTCGCTCGCACCTCGCTCGCGCCAGCCCTTCCCGCTCTTCTGTTCTCGCTCTATTTGCCCCGCTGACTGCTGGCCTCGCCAGCTTTGCCAGTCTTACGT
CTCTGCCGCCCCCACTCCCGCCCGCGCCCCATCTTCTTGCGCGACTCGCGCCCGCTGGTCCCCCCCTCCTCCTCCCGCGTCCTGCCTGCCCCCTCCTCCTGCTCTCGC
AGGCTCCTTGGCACCCAGGCCGGGAGGCGACGCGCCCAGCCGTCTAAACGGGAACAGCCCTGGCTGAGGGAGCTGCAGCGCAGCAGAGTATCTGACGGCGCCAGGTTG
CGTAGGTGCGGCACGAGGAGTTTTCCCGGCAGCGAGGAGGTCCTGAGCAGCATGGCCCGGAGGAGCGCCTTCCCTGCCGCCGCGCTCTGGCTCTGGAGCATCCTCCT
GTGCCTGCTGGCACTGCGGGCGGAGGCCGGGCCGCCGCAGGAGGAGAGCCTGTACCTATGGATCGATGCTCACCAGGCAAGAGTACTCATAG

B

Species	TRE	Position (strand)	Score	P-value	Site sequence
Homo sapiens	DR4	-760(-)	0.771606	0.0280176	GGATCAcctgAGGTCA

Supplementary Figure 11: Localization of the putative TRα1-binding site on the human WIF1 promoter. (**A**) A 5 Kb region upstream of the WIF1 start site was analyzed using the NUBISCAN software, revealing one putative binding sites. The sequence highlighted in bold show the arrangement of the two half-sites as a typical DR4. The first exon is in red, and the first coding ATG (in bold) is underlined. (**B**) Results obtained by NUBISCAN summarizing the score, the *P*-value and the sequence of the putative DR4.

Supplementary Table 1: Circulating serum levels of the hormones T3and T4 (pmol/l) in 10/12-month old animals of different genotype

	Free T3	Free T4
**WT** (*N* = 5)	7.23 ± 3.2	19,56 ± 8.7
**Apc$^{+/1638N}$** (*N* = 8)	8.12 ± 1.2	21.24 ± 4.5
***vil*-TRα1/Apc$^{+/1638N}$** (*N* = 7)	7.42 ± 3.7	18,56 ± 6.3
**TRα$^{0/0}$/Apc$^{+/1638N}$** (*N* = 8)	9.56 ± 2.87	22.67 ± 3.32

Supplementary Table 2: Differentially expressed genes between *vil*-TRα1/Apc and Apc adenocarcinomas. See Supplementary_Table_2

Supplementary Table 3: Analysis of differentially expressed genes by RTqPCR

Wnt

Gene symbol	Regulation by microarray	Fold change	P-value	Validation by RTqPCR
Acvr1c	UP	5.2838616	0.000055953	Yes
Acvr2a	UP	2.4383454	0.002955291	Yes
Dvl3	UP	4.580133	0.000216257	Yes
Frzb	DOWN	-3.6924949	0.001407467	Yes
Fzd5	UP	3.4337366	0.000718328	No
Fzd6	DOWN	-7.5305276	0.000133650	Yes
Nr5a2	UP	3.385896	0.000003153	Yes
Mmp7	DOWN	-50.217285	0.000040614	Yes
Rarb	DOWN	-9.686127	0.000002355	Yes
Rarg	DOWN	-3.0901685	0.000283810	Yes
Smo	DOWN	-3.0170665	0.000001098	Yes
Sox1	UP	3.5006268	0.000061348	Yes
Sox4	DOWN	-21.243048	0.000005287	Yes
Sox7	DOWN	-8.95734	0.002229178	Yes
Sox9	DOWN	-35.4406	0.000030398	Yes
Sox17	DOWN	-55.627438	0.000121541	Yes
Sox21	DOWN	-23.753094	0.000000202	Yes
Src	UP	3.7255604	0.002964700	Yes
Wif1	DOWN	-1371.4115	0.000005095	Yes

Notch

Gene symbol	Regulation by microarray	Fold change	P-value	Validation by RTqPCR
Cntn1	UP	4.9028287	0.000051620	Yes
Dtx1	UP	22.044329	0.005482353	Yes
Hes5	UP	16.803392	0.003187330	Yes
Hey1	DOWN	-7.8807583	0.000398719	No
Jag2	DOWN	-8.332342	0.000051345	Yes
Notch1	UP	7.1939216	0.001253423	Yes
Notch4	DOWN	-4.1032467	0.000488565	Yes
Numb	UP	2.177536	0.000599885	Yes

TH/-TR/NHR signaling

Gene symbol	Regulation by microarray	Fold change	P-value	Validation by RTqPCR
Rarb	DOWN	-9.686127	0.000002355	Yes
Rarg	DOWN	-3.0901685	0.000283810	Yes
Nr5a2	UP	3.385896	0.000003153	Yes

Strongly regulated

Gene symbol	Regulation by microarray	Fold change	P-value	Validation by RTqPCR
Cxcr5	UP	627.18054	0.009746668	Yes
Aicda	UP	233.39041	0.003887780	Yes
Expi	DOWN	-1749.5175	0.002538855	Yes
Anxa10	DOWN	-2380.4036	0.000134514	Yes
Spp1	DOWN	-263.31964	0.000475193	Yes
Ltf	DOWN	-126.68741	0.000930259	Yes
Serpina3n	DOWN	-22.09983	0.000395902	Yes
Tff1	DOWN	-859.4737	0.003658559	Yes
Trim29	DOWN	-31.6185	0.000367182	Yes
Wif1	DOWN	-1371.4115	0.000005095	Yes

Supplementary Table 4: Differentially expressed genes present within the network "Cellular growth, Cell proliferation and Cancer"

Gene symbol	Gene Name	P-value	Fold Change	Location	Family
Pcp4	Purkinje cell protein 4	9.0323704E-10	12.162	Cytoplasm	other
Rit2	Ras-like without CAAX 2	5.575777E-7	7.573	Plasma Membrane	enzyme
Ly6H	Lymphocyte antigen 6 complex, locus H	1.4889147E-7	5.568	Plasma Membrane	other
Dvl3	Dishevelled, dsh homolog 3 (Drosophila)	1.7458719E-5	4.580	Cytoplasm	other
Myo1A	Myosin IA	1.8318578E-5	4.229	Cytoplasm	peptidase
Tspan2	Tetraspanin 2	1.9438273E-8	2.682	Extracellular Space	other
Tspan3	Tetraspanin 3	9.76963E-4	2.232	Plasma Membrane	other
Trappc8	Trafficking protein particle complex 8	1.247536E-4	2.217	Cytoplasm	transporter
Pcdh11X/Pcdh11Y	Protocadherin 11 Y-linked	2.0737643E-5	2.179	Plasma Membrane	other
Wif1	WNT inhibitory factor 1	3.4672276E-8	-1371.41	Extracellular Space	other
Sfrp2	Secreted frizzled-related protein 2	2.767383E-4	-26.721	Plasma Membrane	transmembrane receptor
Dppa2	Developmental pluripotency associated 2	5.4807522E-8	-26.327	Unknown	other
Nkd1	Naked cuticle homolog 1 (Drosophila)	6.1196306E-7	-13.087	Unknown	other
Wnt3	Wingless-type MMTV integration site family, member 3	2.309296E-6	-10.934	Extracellular Space	other
Trpv4	Transient receptor potential cation channel, subfamily V, member 4	1.2442643E-4	-7.394	Plasma Membrane	ion channel
Tnfaip8l1	Tumor necrosis factor, alpha-induced protein 8-like 1	8.699543E-8	-6.543	Unknown	other
Pfkl	Phosphofructokinase, liver	3.0679496E-6	-5.338	Cytoplasm	kinase
Trim13	Tripartite motif containing 13	7.840267E-4	-4.344	Cytoplasm	enzyme
Ano1	Anoctamin 1, calcium activated chloride channel	5.711523E-6	-4.073	Plasma Membrane	ion channel
FrzB	Frizzled-related protein	2.5567264E-4	-3.692	Extracellular Space	other
Ctxn1	Cortexin 1	7.485673E-6	-3.510	Unknown	other
Myo1B	Myosin IB	1.3866271E-4	-3.333	Cytoplasm	other
C15orf23	Chromosome 15 open reading frame 23	4.0513364E-6	-3.049	Unknown	other
Csnk1E	Casein kinase 1, epsilon	1.1423813E-7	-3.006	Cytoplasm	kinase
Tspan6	Tetraspanin 6	3.1708973E-6	-2.729	Plasma Membrane	other
Plod3	Procollagen-lysine, 2-oxoglutarate 5-dioxygenase 3	1.314971E-6	-2.531	Cytoplasm	enzyme

Supplementary Table 5: Analysis of tumors in animals of different age and genotype

AGE: 6-MONTH				
**Genotype**	**Mice** ***N***	**Incidence** ***N*-%**	**Tumors/animal Mean ± SD**	**Tumor range absolute *n*° of tumors**
**$Apc^{1638N/+}$**	3	3/3 100%	1.33 ± 0.58	1–2
**$TR\alpha^{0/0}/Apc^{1638N/+}$**	3	0/3 0%	0^{*}	0

AGE: 8-MONTH				
**Genotype**	**Mice** ***N***	**Incidence** ***N*-%**	**Tumors/animal Mean ± SD**	**Tumor range absolute *n*° of tumors**
**$Apc^{1638N/+}$**	4	4/4 100%	1.25 ± 0.50	1–2
**$TR\alpha^{0/0}/Apc^{1638N/+}$**	5	0/5 0%	0^{**}	0

AGE: 12–15 MONTH				
**Genotype**	**Mice** ***N***	**Incidence** ***N*-%**	**Tumors/animal Mean ± SD**	**Tumor range absolute *n*° of tumors**
**$Apc^{1638N/+}$**	6	6 100%	2.33 ± 0.52	2–3
**$TR\alpha^{0/0}/Apc^{1638N/+}$**	9	9/9 100%	$1.56 \pm 0.53^{**}$	1–2

$^{*}P < 0.05$ and $^{**}P < 0.01$ compared with Apc mice, by Student *T*-test.

Supplementary Table 6: Primers used for different analyses. See Supplementary_Table_6
